# Humoral Epitope Spreading in Autoimmune Bullous Diseases

**DOI:** 10.3389/fimmu.2018.00779

**Published:** 2018-04-17

**Authors:** Dario Didona, Giovanni Di Zenzo

**Affiliations:** ^1^Clinic for Dermatology and Allergology, University Hospital Marburg, University of Marburg, Marburg, Germany; ^2^Molecular and Cell Biology Laboratory, Istituto Dermopatico dell’Immacolata (IDI)-IRCCS, Rome, Italy

**Keywords:** autoantibody, antigen, epitope, epitope spreading, pathogenesis, specific therapy, desmoglein, BP180

## Abstract

Autoimmune blistering diseases are characterized by autoantibodies against structural adhesion proteins of the skin and mucous membranes. Extensive characterization of their autoantibody targets has improved understanding of pathogenesis and laid the basis for the study of antigens/epitopes diversification, a process termed epitope spreading (ES). In this review, we have reported and discussed ES phenomena in autoimmune bullous diseases and underlined their functional role in disease pathogenesis. A functional ES has been proposed: (1) in bullous pemphigoid patients and correlates with the initial phase of the disease, (2) in pemphigus vulgaris patients with mucosal involvement during the clinical transition to a mucocutaneous form, (3) in endemic pemphigus foliaceus, underlining its role in disease pathogenesis, and (4) in numerous cases of disease transition associated with an intermolecular diversification of immune response. All these findings could give useful information to better understand autoimmune disease pathogenesis and to design antigen/epitope specific therapeutic approaches.

## Introduction

Autoimmune diseases are caused by dysregulation of the immune system or arise as a consequence of microbial infection (1–3). In human, many antigens have been identified in patients and several studies have demonstrated that autoantigens, autoantibody, or related autoreactive lymphocytes can transfer autoimmune disease in animal models. Autoantigens are often clustered on the basis of tissue-specific expression or their structural organization. Several studies conducted in animal models and patients with autoimmune disease suggest that epitope spreading (ES) is the probable explanation for this clustering of specificities in human autoimmune diseases. The ES is an important component of the protective immune responses that acts to enhance its efficiency.

The dynamics of autoimmune response has been investigated both in patients and in animal models. However, although several animal models of autoimmune disease have demonstrated the functional value of ES, in patients limited data are available. A possible explanation could be the early occurrence of ES before the diagnosis and the inhibitory effect of therapy on the autoimmune response.

Autoimmune bullous diseases are organ-specific diseases of the skin and mucous membranes characterized by circulating and tissue bound autoantibodies to structural proteins that maintain cell–cell and cell–matrix adhesions. Studies on prospective cohort of patients have allowed to characterize the dynamics of humoral response and have provided in some studies and reports evidence of the functional role of ES.

To study ES can give a better insight into the autoimmune response elucidating: (i) the initiation and progression of the disease and (ii) the meaning of the autoantigen clustering and specific reactivity profile in patients. Furthermore, to understand the possible influence of ES in autoimmune progression and to gain knowledge of relevant autoantigen and epitopes could be crucial for diagnosis and for designing antigen-specific treatments.

Here, we summarize the significance and mechanism of ES and review current literature on humoral ES in the autoimmune bullous diseases that are paradigmatic autoantibody mediated disorders.

## ES: Definition and Significance

Epitope spreading represents the process of diversification of B and/or T-cell response from the initial dominant epitope to a secondary epitope over time. To expand the antigenic epitopes corresponds to optimizing the Ag recognition, to enhancing the neutralization function of antibodies and, in general, to contributing to the efficiency of the immune response.

Epitope spreading that occurs within a single antigen or involves different antigens is termed intramolecular and intermolecular ES, respectively. The intramolecular ES consists of the diversification of immune response in the same autoantigen; the intermolecular ES commonly involves different antigens of a single macromolecular complex or that colocalize in the same anatomical site.

The reactivity that spreads from a single autoantigen to multiple antigens through cross-reactivity is not authentic ES. However, the cross-reactivity could be an initial step to ES. In systemic lupus erythematosus (SLE), autoreactivity to three ribosomal P protein (P_1_, P_2_, and P_3_) is due to a cross-reactive C-terminal epitope present in all three molecules (4). Nevertheless, additional ES phenomenon has been described (5). In this context, the cross-reactivity at the base of molecular mimicry phenomenon can initiate the spreading of an autoimmune response in genetically susceptible hosts. In multiple sclerosis (MS), the initial inflammatory response to a virus infecting the central nervous system can be the triggering factor that induces ES toward autoantigens (6). Similarly, patients with infectious mononucleosis possess circulating IgM to p542, a hematopoietic cell antigen that cross-reacts with Epstein–Barr virus (EBV) nuclear antigen. Afterward, they develop also IgM autoantibodies to p554 that do not cross-react with EBV antigens and that probably arise through ES (7, 8).

In autoimmune response against molecular targets, ES can vary between patients and sometimes reveals a predictable hierarchy. The hierarchy of dominant and secondary epitopes is due to a differential protein processing and presentation, MHC restriction, and to the availability of epitope-specific T and B cells, taking into account central and peripheral tolerance mechanisms.

The predictable sequential ES cascade can present a clinical value. In a subset of patients with mild SLE and relevant skin involvement, autoantibody responses are directed only to the 60 kDa RoAg. In contrast, many patients with primary Sjogren’s syndrome and SLE possess IgG autoantibodies for both Ro and La Ag (9, 10).

The functional value of ES in disease development and progression has been clearly demonstrated in immune response in animal models with autoimmune disease. In relapsing experimental autoimmune encephalomyelitis (EAE), an experimental animal model for MS, the contribution to the pathogenesis and specifically to the relapsing clinical episodes of T cell response against epitopes released as a result of tissue damage *via* ES is evident. Specifically, emerging responses to myelin epitopes are clearly able to mediate disease relapse in this mouse model (11, 12). The measles virus infections in Lewis rats are a further example of the causative role of ES in disease induction. This infection induces inflammatory demyelinating lesions in central nervous system. T cells from rats have been reported to proliferate in response to myelin basic protein (MBP) and no cross-reactivity between MBP and virus was demonstrated (13, 14). T cells activated with MBP and adoptively transferred in recipient rats induce lesions resembling those of EAE model (15). Interesting data on functional ES are also provided in non-obese diabetic (NOD) mice, a model of type 1 diabetes where destruction of pancreatic β cells is mediated by CD4 and CD8 T cells specific for numerous epitopes expressed on insulin (Ins) and other autoantigens. Using NOD splenocytes coupled with intact Ins and several additional diabetogenic epitopes Prasad et al. have demonstrated that Ins B9–23 is a dominant initiating epitope, but autoimmune responses to Ins epitope(s) distinct from Ins B9–23 are driven by ES (16). In this context, the progression to overt disease is associated with responses to epitopes distinct from the initiating B9–23 region (16).

However, a pathological role for ES was not always demonstrated in human diseases. Limited data on the functional relation between ES, clinical severity, and disease pathogenesis have been reported. One reason may be that published studies were small in scale (17–19) or that at the time of the diagnosis the ES phenomenon might already have occurred (20, 21). In addition, since treatments are based on inhibition of autoimmune response ES, could be negatively modulated. Indeed, some interesting data on functional value of ES are reported in patients affected by autoimmune blistering diseases. In bullous pemphigoid (BP), the ES phenomenon seems to be associated with disease severity at diagnosis (22). Furthermore, several reports describe a transition from a certain bullous disease to another one due to an intermolecular ES phenomenon that involves a different disease specific antigen. Maeda et al. described a patient who developed BP 12 years after pemphigus foliaceus (PF) being diagnosed (23). Another example of ES occurred during the progression to SLE. Patient autoantibody profiles showed a target diversification over time. Specifically, anti-nRNP-A antibodies bind to the N-terminus of the protein more frequently in later stages when compared to the diagnosis suggesting a role of this diversification in the progression of autoimmune disease (24).

Finally, further evidence of a functional ES in humans is associated with transplantation of allografts. In several cases, ES of the host response to the allograft and organ allograft rejection are clearly correlated (25, 26).

## Mechanism of ES

Immune responses are characterized by the immunodominance of epitopes within antigens and a great diversity of T- and B-cell epitope specificity. The broadening of the immune response in autoimmune diseases is induced by tissue damage and inflammation, endocytic processing, antigen presentation, and somatic hypermutation (SHM).

There are two major mechanisms at the base of B cell ES in autoimmune diseases: the first is independent of a physical association of antigens while the second is dependent (27). In the “independent” mechanism, tissue damage inflammation and cytokines induce T cells to recognize cryptic epitopes and activate B cells. On the other hand, a “dependent” response relies on the activation of T and B cells by processing and presentation of physical associated antigens.

### Mechanism Independent of Physical Association of Antigens

The development of secondary epitopes in the initial autoantigen or in different autoantigens can depend on the release of antigens or the disclosure of part of antigens during a chronic autoimmune or inflammatory response. In this context, a chronic tissue damage can induce the activation and recruitment of autoreactive lymphocytes specific for epitopes, which are distinct from and non-cross-reactive with the disease-inducing epitope.

An example is the spreading from viral to self epitopes that is shown to play a pathological role in several virus-induced autoimmune disease models (28). A persistent infection can cause the activation of microorganism-specific T cells which mediates tissue damage and release of self peptides (29). Moreover, the induced inflammation can also result in an increased infiltration of T cells at the site of infection and a non-specific activation of self-reactive T cells. Another possible scenario during microbial infection is driven by the IFN-γ secreted by both activated T cells and infected tissue cells. This cytokine can activate antigen presenting cells (APC) and lead to the engulfment of self-antigens. Increased protease production and different processing of captured self-antigens can result in presentation of cryptic self-epitopes that possibly activate autoimmune T and B cells (29). One example of molecular mimicry involves the relation between EBV infection and the development of SLE. The EBV nuclear Ag-1 (EBNA-1) contains a peptide sequence that closely resembles a region on the Smith Ag (Sm) targeted by autoantibodies in SLE patients. Immunization of rabbits with EBNA-1 peptide leads to development of cross-reactivity to Sm antigen that spreads toward an immune response to Sm and nRNP complexes (30). A prospective study on BP patients showed that the autoimmune response starts with autoantibodies specific for an extracellular antigen and then spreads to an intracellular one, suggesting that tissue damage and inflammation play a role in the dynamics of the response (22).

### Mechanism Dependent of Physical Association of Antigens

Autoantigens are frequently part of multiantigen complexes. T cells specific for one epitope of an antigen can activate B cells that are specific for other different antigens of the complex, allowing production of autoantibodies even against antigens/epitopes not originally targeted by the immune response (31). In this case, B cells directed against disease-initiating epitopes mediate antigen-specific uptake of large protein complexes leading to the efficient presentation of self-epitopes from distinct proteins. Immune responses are initiated by presentation of immunodominant epitopes to CD4 T cells by APC such as dendritic cells. The primed CD4 helper T cells then activate antigen-specific B cells. The APC function of B cells induce an increased uptake of antigen that leads to a stimulation of CD4 T cell also by peptides that weakly bind MHC class II molecules. In this context, the processing of antigens is directed by the specificity of the immunoglobulin receptor on B cells. In fact, the epitope bound by the immunoglobulin is protected from proteolysis, and this alters the array of peptides presented to naive CD4 T cells (31). An example of ES dependent of physical association of antigens is the development of autoantibodies to multiple components of the La/Ro ribonucleoprotein complex in SLE and SS. The initiation of an immune response on a single component is at the base of the appearance of mixed autoantibody patterns in these systemic autoimmune diseases (32). The splicesome consists of multiple proteins and nucleic acids associated with each other. Thus, immunization with various SmD peptides leads to the development of autoantibodies not only against regions of the SmD protein but also against the U1-associated ribonucleoprotein (A-RNP) and induces lupus-like symptoms in a mouse model (33).

The mechanism at the base of inter- and intramolecular ES dependent on physical association of antigens can be associated with endocytic processing and SHM. After binding to B cell receptors, the antigen is endocytosed, cleaved and loaded into a compatible MHC class II complex on the cell surface (34). This process can allow the presentation of previously unrecognized epitopes or, in case of endocytosis of multi-antigen complex, the presentation of novel antigen (27, 35). On the other hand, after B cells activation in response to Ag, they undergo SHM in germinal centers. IgV gene experiences single nucleotide substitutions at a frequency of about 10^−3^ per base pair in each B lymphocyte generation (36). Thereafter an affinity maturation process allows a selection of cells with a higher affinity for the Ag. In the context of cell selection, intramolecular ES can occur during the selection of B cells with higher affinity for a different epitope of the Ag or also different Ag (37). Autoantibodies specific for dsDNA can be generated through SHM in developing SLE (38).

Finally, several factors that could influence the ES in the physically linked antigens have been described. In SLE, the strength of non-covalent interactions between Ku antigen and the DNA protein kinase catalytic subunit (p350) is stabilized by autoantibodies that may enhance ES to the p350 antigen (39). In addition, antibody binding of autoantigens may mask some epitopes during antigen presentation or also facilitate presentation of poorly tolerized epitopes (40, 41). In organ-specific autoimmunity, the anatomical isolation of antigens is presumably associated with a less efficient central or thymic T-cell tolerance requiring intrathymic antigen presentation during development of the T-cell repertoire. This partial central tolerance to tissue specific antigens could facilitate the ES. Finally, ES may also be determined by MHC genes, as observed in the experimental model of La/Ro autoimmunity (27).

## Humoral ES in Autoimmune Bullous Diseases

Autoimmune bullous diseases of the skin and mucosae constitute a large group of diseases, including BP, pemphigus vulgaris (PV), PF, paraneoplastic pemphigus (PNP), epidermolysis bullosa acquisita (EBA), linear IgA bullous disease, dermatitis herpetiformis (DH), mucous membrane pemphigoid (MMP), lichen planus pemphigoid (LPP), and others. Their clinical presentation is polymorphic and the pathogenesis is mainly associated with autoantibodies targeting distinct components of the basement membrane zone (BMZ) and desmosome of stratified epithelia. These autoantigens represent structural proteins important for maintenance of epidermal and dermoepidermal integrity. Over the past few decades, identification of autoantigens and relative autoantibodies has improved understanding of the pathogenesis laying the foundation for the study of the dynamics of humoral ES.

### Bullous Pemphigoid

**Figure 1:**
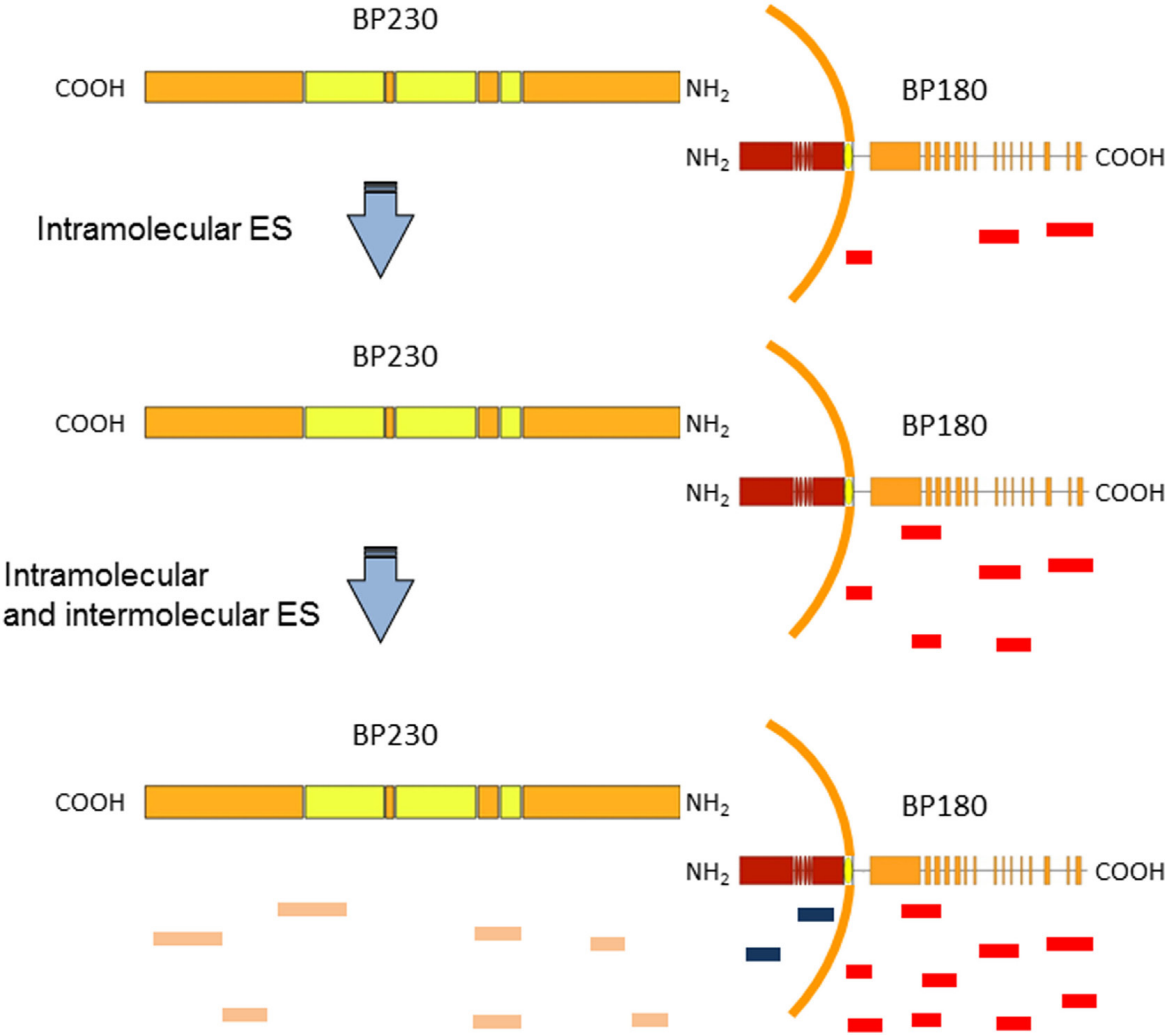
Epitope spreading (ES) in bullous pemphigoid (BP) patients. A schematic representation of BP antigens (BP180 and BP230). Findings support the idea that IgG recognition of the BP180 ectodomain is an early event in BP disease, followed by variable intramolecular ES (red and blue lines in BP180 ectodomain) and intermolecular ES events (orange lines), which likely shape the individual course of BP (22, 58).

Bullous pemphigoid, the most frequent autoimmune bullous disease, typically affects the elderly and is associated with a significant morbidity and mortality. The cutaneous manifestations of BP are protean. In the prodromal, non-bullous phase, patients complain of severe itch sometimes accompanied by eczematiform, papular, and or urticarial lesions. In the bullous stage, vesicles and bullae develop on apparently normal or erythematous skin. Involvement of the oral cavity is observed in 10–30% of cases (42). BP is associated with autoantibody to BP antigen 1 and 2 (BPAG 1 and BPAG2) also known as BP230 and BP180, two components of junctional adhesion complexes in human skin. The diagnosis is based on clinical evaluation and detection by direct immunofluorescence (DIF) of IgG and/or complement deposition along the BMZ in a linear pattern. Serological tests such as indirect immunofluorescence (IIF) and antigen-based ELISAs have a confirmatory value (42). The vast majority of BP sera react with an immunodominant extracellular domain of BP180, termed non-collagenous 16A (NC16A) domain (43, 44). The pathogenic relevance of autoantibodies to NC16A is supported by several experimental evidences (43, 45–47). Although *in vitro* and *in vivo* animal models have shown that IgG autoantibodies to BP180 are pathogenic, the role of anti-BP230 antibodies is only partially clear (48, 49). Several studies in BP patients demonstrated that, in addition to the NC16A domain, other epitopes of BP180 and BP230 are targeted by both autoaggressive B and T cells (50–57). To study the dynamics of IgG reactivity against different BP180 epitopes, Di Zenzo et al. have employed a mouse model in which skin obtained from transgenic mice expressing human BP180 was grafted on the back of wild-type mice. The grafting induced an immune response to BP180 perfectly located in its natural molecular context (58). Interestingly, the IgG reactivity with extracellular epitopes preceded IgG recognition of intracellular domain. Indeed, the spread of humoral immune responses in this graft model was exclusively target specific and the kinetics of graft loss is completely different from alloantigen-related graft rejection previously described in this model (59). A possible interpretation of these data is that antibodies directed against extracellular epitopes of BP180 may have induced tissue damage with consequent exposure of intracellular epitopes (Figure 1). These findings were in accord with a previous study by Di Zenzo et al. on the analysis of the humoral response in a large cohort of BP patients. Reactivity results obtained on a vast array of BP230 and BP180 epitopes demonstrated that the IgG recognition of intracellular epitopes was already present at an early stage of the disease (52). Data from mouse model and BP patients raise the possibility that the development of IgG against intracellular epitopes or antigens may correlate with the initial phase of the disease when the tissue damage starts occurring (Figure 1).

In order to assess the evolution of IgG autoantibodies, a multicenter prospective study in 35 BP patients over a 12-month observation period was performed (22). ELISA and immunoblotting (IB) assays were utilized to assess circulating autoantibodies to BP180 and BP230 peptides. In particular, ES events were detected in 17 of 35 patients and preferentially occurred at an early stage of the disease. The functional role of ES was supported by its significant relation to disease severity at diagnosis. Moreover, in line with data obtained by Di Zenzo et al. in mice grafted with human BP180, in three patients the spreading of IgG reactivity to intracellular epitopes of BP180 and BP230 (an intracellular antigen) was preceded by recognition of the BP180 ectodomain (Figure 1).

All these findings suggest that IgG recognition of the BP180 ectodomain is an early event, followed by intra- and intermolecular ES events, which shape the individual course of BP. In addition, these data could give useful information for design therapeutical approaches. In particular, to deplete pathogenic autoantibodies, other pathogenic region of BP180 (in addition to NC16A) should be considered as possible therapy using decoy peptides to block autoantibody binding *in vivo*. However, this approach may require early and massive administration of several different peptides.

Several cases of automimmune bullous disease patients indicate that an ES phenomenon often leads to a disease transition. Recently, Sardy et al. reported an interesting case of an 18-year-old Caucasian woman, affected by BP, who showed clinical and laboratory transition to MMP 5 years after the BP diagnosis (60). The patient developed a severe esophageal stenosis, that was not present at the time of the BP diagnosis. DIF detected linear IgG and C3 fluorescence along the mucosal side of the BMZ. Furthermore, IgG autoantibodies directed against the immunodominan epitope of BP180 (NC16A domain) and the BP230 COOH-terminal were identified. Laminin 332 ELISA was positive. Immunoblot studies showed presence of IgG4 autoantibodies to laminin 332, and both IgA and IgG against desmoglein 3 (Dsg3). Collagen VII (Coll VII) autoantibodies were detected neither by ELISA nor by immunoblot. At the time of BP diagnosis, no evidence of MMP have been detected, and anti-laminin 332, autoantibodies were not tested. In addition, retrospective analysis of the serum samples showed no reactivity. After the BP diagnosis, the patient was started on topical steroid therapy and a short course of oral prednisolone obtaining a partial BP remission. The authors postulated that insufficient immunosuppressive treatment of BP might facilitate ES, and lead to a more complex clinical course (60). Another interesting case was reported by Kasperkiewicz et al. In a 70-year-old man with hemorrhagic blisters, widespread crusted erosions, and the immunopathological characteristics of anti-p200 (γ1 laminin subunit) pemphigoid, treatment with doxycycline, topical corticosteroids and immunoadsorption led to rapid clinical remission. Nineteen weeks later, a relapse occurred with an altered clinical phenotype together with autoantibodies against both p200 and NC16A suggested a functional role of ES in the disease transition (61). Autoimmune bullous diseases often develop in patients with psoriasis. BP was the most prevalent followed by antilaminin γ1 pemphigoid (62). A possible explanation of the development of BP in psoriatic patients is provided by previous studies that have shown that laminin 1 and laminin α1 within BMZ are disrupted in both involved and uninvolved psoriatic lesions (63, 64). In general, the damage to BMZ in patients with psoriasis may induce the development of several antibodies such as anti-laminin γ1 and -BP180. However, although a Taiwanese population study disclosed that psoriasis occurred significantly more in patients with BP than that in the control group, large population studies in each country are necessary to support this association (65).

### Pemfigus Vulgaris

Pemphigus includes a group of potential life-threatening autoimmune blistering diseases of the skin and mucous membranes. These diseases are characterized by autoimmune responses mainly directed against two transmembrane components of desmosomes, Dsg3 and Dsg1 (66). Similar to other cadherins, Dsg3 and Dsg1 comprise five extracellular subdomains of approximately equal size (EC1–EC5). Several distinct forms of pemphigus have been reported (PV, PF, PNP, and others); the most common is PV. In PV, the critical role of autoantibodies to Dsg3 is largely demonstrated (67–71). The diagnosis clinical and histopathological findings are supported by demonstration of the IgG deposition on the surface of the epithelial cells by DIF. Detection of circulating autoantibodies against the cell surface by IIF and/or to recombinant Dsgs by ELISA can confirm the diagnosis (69). Usually, the oral mucosa is first involved and then blisters arise on the whole body. In the skin, Dsg3 is mainly expressed in the basal and suprabasal layers, while Dsg1 is predominantly expressed in the upper epidermal layers. However, in oral mucosa Dsg3 is highly expressed throughout the epithelium, while Dsg1 is less expressed. According with “Dsg compensation” theory, the Dsg3/Dsg1 autoantibody profile is at the base of different clinical variants of pemphigus depending on differential expression pattern of Dsg1 and Dsg3 (72). More specifically, in PV patients antibodies to Dsg3 cause mucosal disease due to lack of compensation by Dsg1, while they don’t induce cutaneous disease because of compensation by Dsg1. In PV, patients who acquire antibodies to Dsg1, compensation is no longer possible, resulting in cutaneous as well as mucosal disease (67, 73, 74). On the other hand, in PF antibodies to Dsg1 cause cutaneous disease, while they cannot cause mucosal disease, because Dsg3 is present at high levels throughout the epithelium (75). In accord with this theory are the results obtained in a mouse model by Ding et al. The authors showed that the passive transfer of PV anti-Dsg1 and -Dsg3 antibodies were able to generate cutaneous lesion while anti-Dsg3 antibodies alone were not (73).

**Figure 2:**
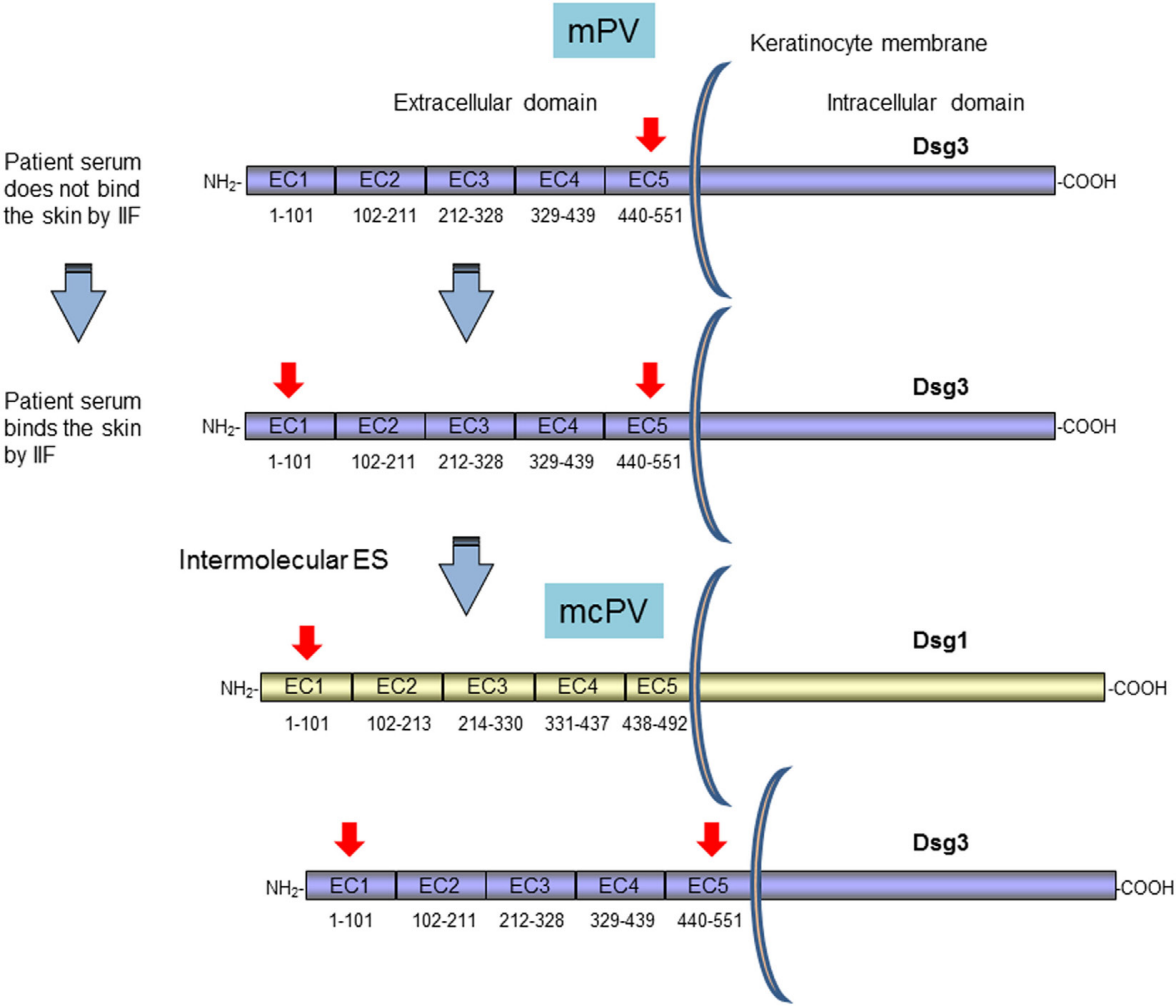
Clinical transition between mucosal pemphigus vulgaris (PV) to mucocutaneous PV as proposed by Salato et al. Survey and schematic representation of the antigenic regions recognized by autoantibodies of a representative PV patient with only mucosal involvement (mPV) that shift clinical phenotype to mucocutaneous one (mcPV) (76). PV antigens (Dsg1 and Dsg3) with relative subdomains (EC1–EC5) of Dsg1 and 3 and their amino acid sequences are illustrated. Red arrows indicate the regions recognized by patient autoantibodies during the course of the disease (76). The mPV patient at an early stage of disease has autoantibodies specific for ectodomain (EC5) subdomain. At this time the circulating autoantibodies are not able to bind human skin by [indirect immunofluorescence (IIF)]. Several years later, the patient produces autoantibodies directed to EC1 by an intramolecular ES phenomenon. At this stage the autoantibodies start to bind the human skin and an intermolecular ES toward Dsg1 occurred with development of cutaneous as well as mucosal blisters (76).

To investigate the ES phenomenon in PV and to identify the basis of clinical transition from mucosal to mucocutaneous involvement, Salato et al. analyzed sera from PV patients taken at various times during the course of disease (76). A subset of PV patients transitioned from mucosal PV to mucocutaneous PV and their autoantibodies profile were evaluated. One representative patient, with only mucosal involvement at an early PV stage, had autoantibodies specific for C-terminal region of Dsg3 ectodomain in the EC5 subdomain. At this time the circulating autoantibodies were not able to bind human skin by IIF (76) (Figure 2). Several years later, the patient produced autoantibodies directed to EC1 subdomain of Dsg3 demonstrating an intramolecular ES phenomenon. Interestingly, the autoantibodies started to bind the human skin by IIF and an intermolecular ES toward Dsg1 occurred (Figure 2). At this stage, the patient exhibited non-cross-reactive autoimmunity to both Dsg3 and Dsg1 and developed cutaneous as well as mucosal blisters (Figure 2). In this context, it is important to underline that the pathogenic activity of anti-Dsg1 autoantibodies affinity purified from a PV patient serum was previously demonstrated in a mouse model based on antibody passive transfer (77). Previous cases with mucosal involvement and anti-Dsg3 autoantibodies that spread to Dsg1 with cutaneous involvement in addition to mucosal one were also reported (73, 78). A major limitation of study from Salato et al. was the use of Dsg1/Dsg3 swapping domains approach for the epitope mapping of autoantibody reactivity. In fact, although the majority of anti-Dsg3 IgG autoantibodies in PV did not cross-react with Dsg1, in mucocutaneous-type PV, with both anti-Dsg1 and anti-Dsg3, autoantibodies mapping results from this approach, may be less reliable, because of the sequence homology of two cadherins. In addition, the exclusive presence of autoantibody to the C-terminal domain of Dsg3 in PV patients with mucosal lesions appeared in contrast with several studies that showed that the vast majority of PV sera react with the N-terminal portion of Dsg3 and rarely bind the C-terminal one (79, 80). On the other hand, the fact that an intermolecular ES from Dsg3 to Dsg1 and not the presence of cross-reactive antibodies is at the base of clinical phenotype transition in PV was demonstrated by an inhibition assay in PV patients with mucocutaneous lesions (67). However, it could be hypothesized the presence of few cross-reactive not detectable antibodies that shift the reactivity from Dsg3 to Dsg1 possibly followed by an intramolecular ES event in the Dsg1 molecule. Indeed, the isolation from PV patients with Dsg1/Dsg3 cross-reactive monoclonal autoantibodies could corroborate this hypothesis (42, 81).

**Figure 3:**
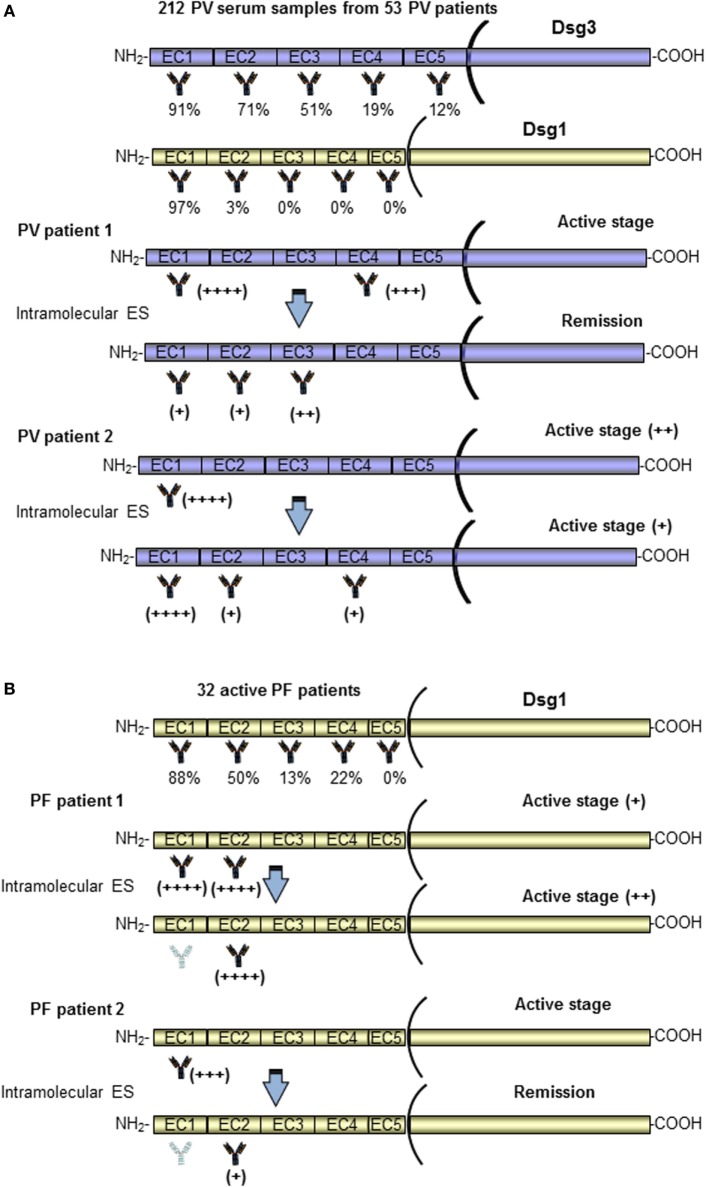
Pemphigus vulgaris (PV) and pemphigus foliaceus (PF) patients present Dsg3 and Dsg1 epitope profiles stable over the disease course. A schematic representation of the antigenic regions recognized by autoantibodies of a PV **(A)** and PF **(B)** patients at diagnosis, with relative percentage of reactivity, and during the disease course (20, 21). Only two patients (for PV and PF) in whom epitope spreading (ES) phenomenon occurred have been shown. In most PV and PF patients, the major Dsg3 and Dsg1 epitopes are localized in the EC1–2 subdomains. In the vast majority of PV and PF patients, the epitope profile remained stable during the course of the disease. Of note, two patients (PV patient 1 and PF patient 2) in active stage reacted to EC1 and shifted with an intramolecular ES to other subdomains (EC2/EC3 and EC2) (20, 21).

In contrast with data from Salato et al., another study using the same approach has reported a stable epitope profile during disease course in four PV patients (80). A further study analyzed ES events in a larger cohort of PV patients (53 patients) at multiple disease stages (20). In this case, a different system based on Dsg3 (or Dsg1)/Dsg2 domain-swapped molecules was employed. In this approach using Dsg2 cadherin as the backbone of the domain-swapped molecules, the epitopes recognized by anti-Dsg3 IgG could be analyzed without the influence of anti-Dsg1 IgG autoantibodies and vice versa, because PV sera showed no reactivity with Dsg2. As previously reported, the major epitopes recognized by PV sera were calcium-dependent and mapped at the N-terminal region of Dsgs (Figure 3A). Interestingly, the recognized region appeared to be unchanged over the course of disease in both anti-Dsg3 mucosal dominant-type PV and anti-Dsg3/Dsg1 mucocutaneous-type PV (20). Intramolecular epitope shift was not evident in the vast majority of PV cases and only two patients of 53 showed an intramolecular ES event in the Dsg3 during the disease course. Specifically, a PV patient (PV1) in active stage reacted to EC1/4 and shifted to EC2/3 during remission, strongly reducing the anti-EC1 reactivity, and another one (PV2) showed a transition from active to moderate disease characterized by a stable anti-EC1 reactivity and the appearance of anti-EC2/4 antibodies (Figure 3A) (20). Independent of disease stage, in most PV patients the major Dsg3 epitopes are localized in the EC1–2 subdomains, while in PV with cutaneous involvement dominant epitopes of Dsg1 are in addition present in EC1 but not in the EC2–5 subdomains (Figure 3A) (20). In contrast to the results of Salato et al., no mucosal dominant-type PV sera reacted only with the EC4 or EC5 subdomain of Dsg3. Further studies with larger cohort of well characterized patients with only mucosal and mucocutaneous phenotype are needed to clarify the relation between recognized epitope and clinical phenotype and to better understand the role of ES in this phenotype transition. All these studies show three major limitations (i) it was impossible to evaluate ES before the onset of disease, (ii) treatment could influence the ES during the course of the disease, and (iii) a possible intradomain ES was not investigated.

In addition to Dsg3 and Dsg1, several other organ-specific non-Dsg autoantibodies in pemphigus patient sera could be involved in an intermolecular ES phenomenon. For example, antibodies against an acetylcholine receptor and pemphaxin that are not pathogenic alone, but may act synergistically with pathogenic anti-Dsg3 antibodies (82–85). Another example are antibodies targeting keratinocyte mitochondria that contribute to the process of acantholysis and could be also involved in ES phenomena associated with the tissue damage induced by pathogenic autoantibodies (85, 86). Other autoantibodies specific for intracellular domains of Dsgs, a calcium pump encoded by ATP2C1, desmocollin 1, BP230, periplakin, E-cadherin, Dsg4, desmoplakin 1, and desmoplakin 2 have been detected in pemphigus patients (69). However, since their pathogenic role has not been demonstrated they could be considered a result of an intermolecular ES phenomenon without any functional role in the disease. According with this possible interpretation are findings from our study on the presence of other non-disease specific autoantibodies in autoimmune bullous disease such as anti-BP180, BP230 and Coll VII autoantibodies in PV patients or anti-Dsg3 autoantibodies in BP. Interestingly, the reactivity against additional non disease-specific antigens was rare and remained stable during disease course, while in the same patients reactivity against disease-specific antigens fluctuated in parallel with disease severity. This atypical reactivity possibly represented an epiphenomenon unrelated to the evolution and progression of the autoimmune disease (87). Further studies are needed to understand the possible role of ES in the generation of these autoantibodies and their role in disease pathogenesis.

In a recent study, Cho et al. have postulated a functional role of ES in the pathogenesis of PV. In particular, they have isolated cross-reactive rotavirus/Dsg3 antibodies from PV patients. A subset of these antibodies had the potential to confer protection against rotavirus infection but also to cause pathogenic effects on skin, suggesting a role of molecular mimicry in the disease pathogenesis (88). In this context, the authors speculate that even in the presence of non-pathogenic and cross-reactive antibodies, the activation of a cross-reactive B cell could stimulate Dsg3-reactive T cells to trigger a broader anti-Dsg3 B cell response, that react to other and possibly pathogenic epitopes leading to PV (88).

### Pemfigus Foliaceus

Pemphigus foliaceus is an autoimmune blistering skin disease characterized by superficial cutaneous erosions. Tissue bound and circulating autoantibodies that react to Dsg1 have shown a pathogenic role in the disease (75, 89) and provide important diagnostic clues in addition to clinical evaluation. The most common subtype is sporadic PF occurring all over the world, whereas endemic PF (EPF) is found in rural areas of Brazil, where the disease is known as fogo selvagem (90–92).

In 2010, Chan et al. have characterized the dynamics of immunoreactivity to various Dsg1 extracellular subdomains in non-endemic PF patients during the course of the disease by using the Dsg1/Dsg2 domain-swapped molecules approach. According to data obtained on PV patients, most of the anti-Dsg1 antibodies bind to the N-terminus region of Dsg1 (Figure 3B), and this reactivity prevails across various activity stages. Only two PF patients lost their EC1 reactivity upon remission and in one case shifting to EC2 (Figure 3B) (21). Thus, in PV such as in PF patients, the Dsg3 and Dsg1 epitope profiles remained stable over the disease course and, because of the rarity of ES, treatments based on the depletion or abrogation of autoantibodies to the N-terminal domains of Dsg3/Dsg1 should be promising (Figure 3B). Another possible involvement of ES is described in a previous study from Ishii et al. that have isolated several pathogenic and non-pathogenic monoclonal antibodies from PF patients (93). Some non-pathogenic antibodies bound to the proprotein of Dsg1, which are thought to be synthesized as intracellular inactive precursor proteins with prosequences that are cleaved to yield mature and extracellular adhesive molecules. Similarly, non-pathogenic autoantibodies from PV patients were also able to bind the precursor of Dsg3 (94, 95). Interestingly, these findings could suggest that PV and PF patients at first develop non-pathogenic antibodies against the intracellular Dsg precursor to which they would not be expected to have tolerance, and in some susceptible patients the antibody response might shift through ES to pathogenic autoantibodies specific for the mature molecule (93–95). Further studies are needed to confirm this hypothesis.

**Figure 4:**
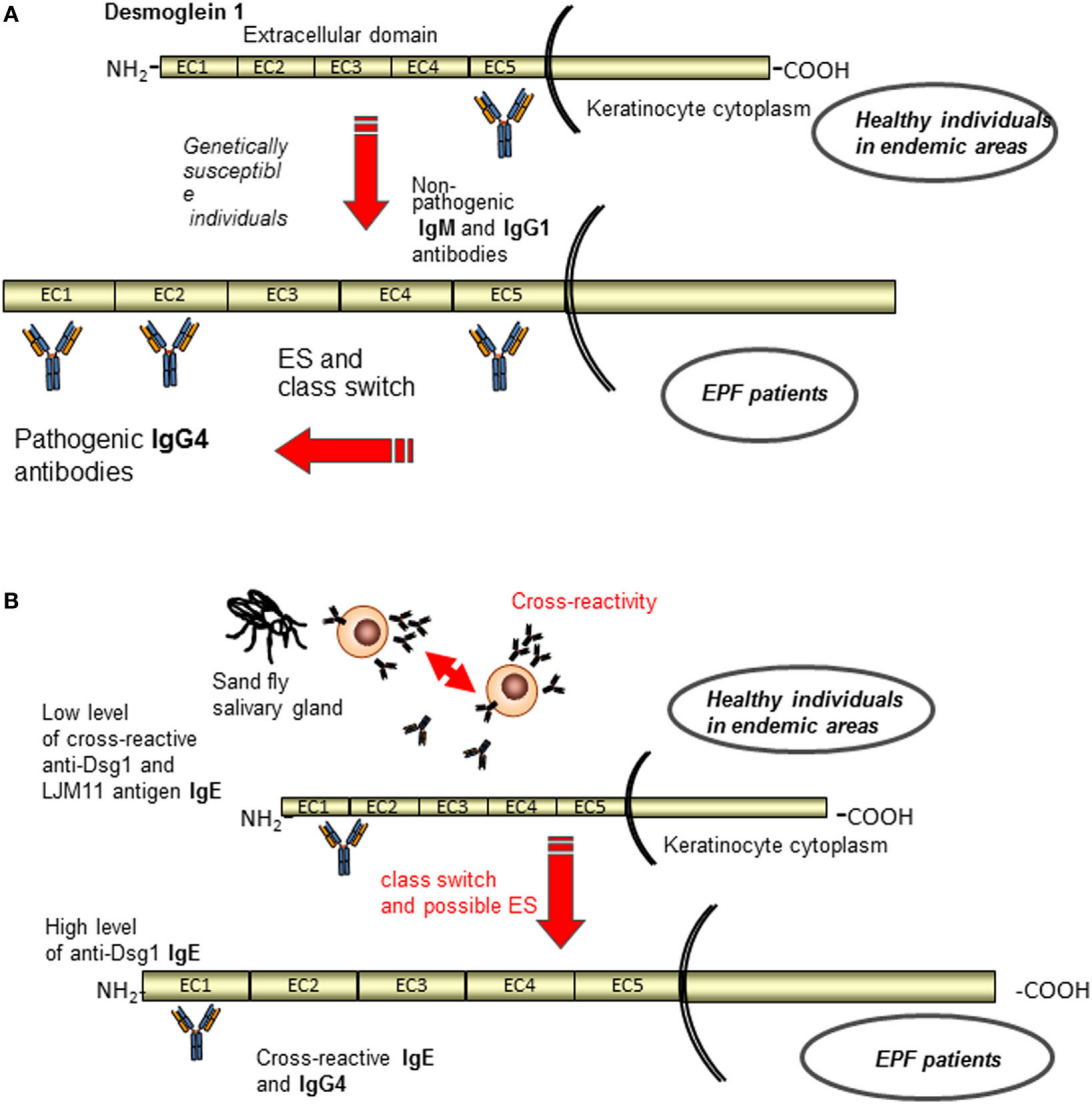
Current etiopathogenetic model for endemic pemphigus foliaceus (EPF). A schematic representation of the antigenic regions recognized by autoantibodies of normal individuals and EPF patients. **(A)** Patients in the preclinical stage and healthy individuals from endemic areas possess IgG1 circulating autoantibodies that recognize non-pathogenic epitopes in the EC5, whereas when the disease developed appeared pathogenic IgG4 antibodies to EC1 and EC2 through class switch and ES (96–98). **(B)** In parallel, antigen mimicry that induces with or without ES an antibody switch from an epitope of salivary gland fly antigen (IgE) to a pathogenic epitope on the EC1 subdomain of Dsg1 (IgG4) is shown (102).

Although the sporadic PF and EPF form share similar immunological and clinical features, the latter presents peculiar characteristics. Specifically, a clinical study suggests that intramolecular ES could be the cause for the development of EPF (96). Patients in the preclinical stage and healthy individuals from endemic areas possessed IgG1 circulating autoantibodies that recognize non-pathogenic epitopes in the C-terminal domain of the Dsg1 ectodomain (EC5), whereas when the disease developed in individuals with certain HLA susceptibility genes pathogenic antibodies directed against the N-terminal region of Dsg1 ectodomain (EC1 and EC2) appeared. Concomitantly a possible antibody subclass switching from IgG1 to IgG4 seemed to occur (Figure 4A) (96–98). In this context, Warren et al. have previously shown that up to 55% of healthy individuals living in endemic areas have low levels of anti-Dsg1 antibodies (97). In addition, a strong relationship has been found between infestation with hematophagous insects, certain HLA susceptibility genes and the occurrence of fogo selvagem, suggesting a role of environmental factors in development of the disease (99). In accord with this idea, Qian et al. have found in patients IgG4 and IgE autoantibodies that cross-reacted with a salivary antigen (LJM11) from sand flies targeting a shared conformational epitope in the EC1-EC2 subdomains (100). Compared to normal controls, individuals before and after onset of fogo selvagem have significantly higher IgE anti-LJM11 and anti-Dsg1 antibodies, suggesting that the IgE antibodies develop before the onset of EPF (100). However, individuals have significantly lower levels of anti-Dsg1 IgE before fogo selvagem onset than after the development of the disease, suggesting that LJM11 might be the initial target of IgE response (101) (Figure 4B). In order to further investigate this cross-reactive immune response and to clarify whether it represents a non-specific activation of the immune system or an antigen-selected response, an antibody phage display library approach was employed. Qian et al. generated libraries comprising only IgG4 subclass from three EPF patients and 14 clonally independent IgG4 monoclonal antibodies were isolated and analyzed (102). Noteworthy, all of these IgG4 monoclonal antibodies were cross-reactive to both Dsg1 and LJM11 and extensively mutated. Furthermore, the revertant monoclonal antibodies, which represent the germline configuration, also recognized both Dsg1 and LJM11 (102). Collectively, these findings suggest that the development of anti-Dsg1 IgG4 antibody in EPF patients could be the result of initial IgE response to the environmental antigen (such as LJM11). In this context, a chronic stimulation of LJM11 antigen and the production of IL10 by the immune system promote the development of pathogenic IgG4 antibodies, which are cross-reactive to both LJM11 and Dsg1 (Figure 4B). Very recently, Evangelista et al. have demonstrated that fogo selvagem is mediated by pathogenic IgG4 autoantibodies against the EC1 subdomain of Dsg1 (103). However, whether the epitope on LJM11 shares its conformational structure with this pathogenic epitope or with a non-pathogenic epitope on Dsg1 that through an intradomain ES could generate pathogenic autoantibodies has not been investigated (103). Thus, putting the evidences all together, IgG4 pathogenic autoantibodies in EPF could be the result of an intramolecular ES event from non-pathogenic autoantibody specific for EC5 subdomain of Dsg1 to a pathogenic one against EC1 subdomain (Figure 4A). In parallel, antigen mimicry that induces with or without ES an antibody switch from an epitope of salivary gland fly antigen to a pathogenic epitope on the EC1 subdomain of Dsg1 has been demonstrated (Figure 4B).

As postulated, for the clinical transition from mucous to mucocutaneous PV an intermolecular ES phenomenon could also be at the base of disease transition. In particular, transition from PV to PF and *vice versa* is a well-documented phenomenon (Table 1). In most cases, PV patient shift to PF, although some cases where PF patients switched to PV have been described (104–120). The mechanism of transition between PV and PF is unclear, and a possible role of ES in this phenomenon has been postulated (Table 1). In cases of PV transforming from PF, anti-Dsg1 autoantibodies were initially present (108, 109). Dsg3 may be released from skin lesions leading to the production of anti-Dsg3 autoantibodies and a phenotype switch. It is unclear if transition from PV to PF is permanent or whether it is due to preferential suppression of Dsg3 antibodies caused by the immunosuppressive therapy (114). Indeed, only in few cases the transition was reported in patients without therapy (104, 113). Rarely, pemphigus and the pemphigoid group of diseases may develop in the same patient (121). Up to date, only four reports about PF to BP transition have been reported (23, 122–124). Usually, the IgG detected in those patients belongs to IgG1 or IgG4. However, Recke et al. reported the presence of IgG3 in their patient. In all the cases described, the transition to BP was associated with a complete disappearance of anti-Dsg autoantibodies highlighting a functional role of ES in these cases (124).

**Table 1:** Cases of pemphigus with clinical shifting between PF and PV.

Reference	Transition	Age	Sex	Initial Ab	Transition period (years)	Ab after transition
Iwatsuki et al. (104)	PV to PF PV to PF	36 58	F F	NP NP	3 1	Dsg 1^a^ NP

Kawana et al. (105)	PV to PF PV to PF	57 46	F F	Dsg 3^a^ Dsg 3^a^	3 3	Dsg 1^a^ Dsg 1^a^

Chang et al. (106)	PV to PF	47	M	NP	4	Dsg 1^a^ Dsg 3^a^

Mendiratta et al. (107)	PV to PF	46	W	NP	NP	NP

Ishii et al. (108)	PF to PV	35	M	Dsg 1^b^	6	Dsg 1^b^ Dsg 3^b^

Komai et al. (109)	PF to PV PV to PF and again to PV	40 38	NP NP	Dsg 1^b^ Dsg 1^a^^,^^b^ Dsg 3^a^^,^^b^	0.5 1	Dsg 1^a^ Dsg 3^a^^,^^b^ Dsg 3^a^^,^^b^ Dgs 1^a^ Dsg 3^a^^,^^b^

Kimoto et al. (110)	PV to PF	77	F	Dsg 3^b^	5	Dsg 1^b^

Tsuji et al. (111)	PV to PF	55	M	Dsg 3^b^	3	Dsg 1^b^

Harman et al. (112)	PV to PF	44	F	Dsg 1^b^ Dsg 3^b^	5	Dsg 1^b^

Toth et al. (113)	PV to PF	28	M	Dsg 1^b^ Dsg 3^b^	2	Dsg 1^b^

Ng et al. (114)	PV to PF PV to PF PV to PF	29 56 45	M M F	NP NP NP	4 3 3	Dsg 1^b^ Dsg 1^b^ Dsg 1^b^

Park et al. (115)	PF to PV	48	M	Negative^a^	5	Dsg 1^b^ Dsg 3^b^

Awazawa et al. (116)	PF to PV	79	F	Dsg 1^b^	1.5	Dsg 3^b^

Pigozzi et al. (117)	PF to PV	90	F	Dsg1^b^	0.7	Dsg3^b^

Lévy-Sitbon et al. (118)	PV to PF	47	M	NP	2.7	NP

España et al. (119)	PV to PF PV to PF	49 35	M M	Dsg 1^a^ Dsg 3^a^ Dsg 3^a^	2 3.8	Dsg 1^a^ Dsg 1^a^ Dsg 3^a^

Ito et al. (120)	PV to PF	57	M	Dsg 1^b^ Dsg 3^b^	0.2	Dsg 1^b^

*^a^Immunoblot*.

*^b^ELISA*.

*Ab, antibodies; PV, pemphigus vulgaris; PF, pemphigus foliaceus; NP, not provided; Dsg, desmoglein*.

### Paraneoplastic Pemphigus

Paraneoplastic pemphigus is a rare autoimmune bullous disease that is always associated with neoplasm (125, 126). Typically, PNP patients show autoantibodies directed against the plakin family, including antibodies against envoplakin, periplakin, desmoplakins I and II, plectin, the hemidesmosomal protein BP180 and BP230 (125, 126), and the protease inhibitor alpha-2-macroglobulin-like-1 (127). Furthermore, antibodies against plakophilin 3, desmocollins 1, desmocollins 3, Dsg1, and 3 have also been detected in PNP (125, 126). Clinically, patients are characterized by the presence of severe mucocutaneous lesions and sometimes respiratory failure, and they are refractory to standard treatment. Diagnosis mainly based on DIF, relies also on the demonstration of autoantibodies in serum, assessed by IB, ELISA, IIF on rat bladder and immunoprecipitation with radioactive keratinocyte extracts. IIF on rat bladder epithelium that expresses plakins and not Dsg is useful for differential diagnosis with pemphigus. However, this approach is not completely reliable considering that some pemphigus patient reacts with plakins in addition to Dsgs (128–131).

Bowen et al. suggested that ES might play a role in the pathogenesis of PNP (132). They described five PNP patients showing clinical and pathological features of lichen planus (LP) (132). Among them, one showed initially negative results by IIF on rat bladder epithelium; however, one year after, positive IIF staining was observed. Furthermore, the same patient showed initially only autoantibodies against alpha-2-macroglobulin-like-1 of 170 kDa and desmoplakin I; after one year, IB detected also desmoplakin II, BP230, and periplakin (132). Therefore, it has been postulated that the lichenoid eruption, which led to necrosis of keratinocytes, might be a mechanism for exposing previously masked antigens to autoreactive T cells, leading to an intermolecular ES that involves other skin antigens. Another PNP case, in which ES might be involved, was reported by Okahashi et al. (133). They described a 41-year-old patient who initially showed only mucosal lesion; however, 3 months after PNP diagnosis, he also developed toxic epidermal necrolysis (TEN)-like cutaneous lesions. IgG against envoplakin and periplakin were detected by IB both at the time of PNP diagnosis and when the patient developed cutaneous lesion. Contrarywise, ELISA detected anti-Dsg1 and three IgG only after the arise of cutaneous lesion. Therefore, the authors proposed that autoimmune response led initially to interface and lichenoid dermatitis, which exposed self-antigens developing humoral autoimmunity mediated by ES (133).

In 2012 cloning monoclonal antibodies from a PNP patient by using phage display approach, the pathogenic activity of anti-Dsg3 autoantibodies has been demonstrated (134). Epitope mapping using domain-swapped Dsg3/Dsg2 showed that these monoclonal antibodies bound conformational epitopes in the EC2 and EC3 domains. In parallel, to study the Dsg3 epitope profile in PNP Ohyama et al. (20) tested sera from 14 patients. Interestingly, in addition to the reactivity against the EC1 subdomain of Dsg3 detected in all patients, 57%, 71%, and 86% of PNP sera reacted with the EC2, EC3, and EC4 subdomain, respectively. Thus, circulating antibodies in PNP had unique epitope profile, although, such as in PV and PF, the most recognized region is the Dsg N-terminal one. These findings were consistent with a previous study (135) and suggested that the typical autoantibody profile may have contributed to their unique clinical and histopathological features.

Several theories have been proposed to explain the association between bullous disease and tumor. One theory proposed that immune response against tumor antigens cross-reacting with epithelial antigen can lead to an autoimmune response against skin and mucous membrane (136). Another theory proposed that the tumors may have caused cytokine dysregulation leading to autoimmunity to Dsgs with subsequent tissue damage and intermolecular ES toward intracellular proteins of the plakin family and or hemidesmosomal proteins. However, although the pathogenic role of anti-Dsg3 autoantibodies has been demonstrated (134), autoantibodies to Dsg3 are not detected in all patients and not always detected at the beginning of the disease onset (133). Another possible explanation is that the cellular damage induced by tumor specific immune response could expose intracellular antigen, such as plakins, and lead to an autoimmune response to the skin and mucous membrane and a possible intermolecular ES toward other antigens such as Dsgs.

Indeed, with the exception of few case reports, the dynamics of humoral response during disease course was not reported in PNP patients and could be the objective of future studies.

### Epidermolysis Bullosa Acquisita

Epidermolysis bullosa acquisita is an acquired, subepidermal bullous disease. The main feature of EBA is autoimmunity to Col VII with consequent reduction of anchoring fibrils (137). On the one hand, a classical “mechanobullous” form has been described, which is characterized by blistering, erosions with milia formation, and scarring over trauma exposed sites (138). On the other hand, several patients with an “inflammatory” form of EBA mimicking BP, characterized by blisters on intertriginous areas that heal without scars, have been reported (138). Clinical diagnosis is confirmed by demonstration of IgG and/or IgA and C3 deposits at the BMZ by DIF. In EBA, IIF performed on salt-split skin (SSS) usually shows binding of antibodies to the dermal side of the blister and circulating autoantibodies bind to Coll VII by ELISA or IB (139). Specifically, EBA is mainly characterized by IgG autoantibodies to the non-collagenous domain (NC1) of Coll VII (137, 139). However, no evidence of intramolecular and intermolecular ES in an active mouse model of EBA was found. Reactivity to additional regions outside Coll VII did not occur in mouse suggesting that ES does not contribute to EBA development in this mouse model (140).

Epitope spreading that involves EBA antigen is reported in sera from patients with SLE that contain autoantibodies to Coll VII (141, 142). Chan et al. reported the case of a 15-year-old patient, who showed IgA and IgG circulating autoantibodies against BP230, as well as against the full-length native form and the recombinant NC1 domain of Coll VII (143). In addition, IgG autoantibodies against laminin 332 and laminin 311 have been detected in the same patient. These findings could be explained by an intermolecular ES phenomenon. Indeed, because the NC1 domain of Coll VII bound to the β3 chain of laminin 332, it was thought that the initial autoimmune reaction against Coll VII led to secondary damage of laminin 332 and other adjacent BMZ component, like laminin 311 and BP230 (144). In addition, several reports of EBA patients who subsequently developed SLE or vice versa have been described and could represent the effect of an ES phenomenon (141, 143, 145–150) (Table 2). Dotson et al. were the first to describe a 19-year-old EBA patient, who developed clinical and serologic evidence of SLE five years after the diagnosis of EBA (145). Cutaneous lesions examinated by electron microscopy before and after the development of SLE showed characteristics previously reported by electron microscopy in patients with EBA. DIF before and after the diagnosis of SLE highlighted linear depositions of immunoglobulin and complement; IIF findings showed no abnormalities.

**Table 2:** Patient showing coexistence of EBA and SLE disease and/or antigens.

Reference	Number of patients	First diagnosed disease	Age and sex	Laboratory findings	Method	Note
Dotson et al. (145)	1	EBA	19, F	ANA (1:2560), anti-U1RNP Ab	–	SLE diagnosis 5 years after EBA diagnosis

Barton et al. (146)	1	BSLE	18, F	IgG against COLVII	IB	–

Gammon et al. (141)	4	1 Pt SLE; 3 Pt BSLE	3 Pt 20–23, 1 Pt 50; 3 F, 1 M	IgG against COLVII	WB	–

Kettler et al. (147)	1	EBA/BSLE?	8, F	ANA (1:2560), anti-U1RNP Ab, anti-Sm Ab	–	–

Boh et al. (148)	3	EBA	34, F	Homogeneous ANA (1:160) anti-dsDNA Ab (1:640), anti-U1RNP Ab	–	SLE diagnosis 2 years after EBA diagnosis
			51, F	Speckled ANA (1:640), low C3, anti-dsDNA Ab (1:40), anti-U1RNP Ab		SLE diagnosis 3 years after EBA diagnosis
			57, F	Speckled ANA(1:640), anti-ds-DNA Ab, anti-U1RNP Ab, anti-Sm Ab		SLE diagnosis 14 years after EBA diagnosis

McHenry et al. (149)	1	SLE	77, M	–	–	EBA diagnosis 6 years after SLE diagnosis

Yoon et al. (150)	1	EBA	38, F	–	–	EBA preceded a dramatic SLE flare with fatal cerebral vasculitis

Chan et al. (143)	1	BSLE	15, F	IgA and IgG against BP230, NCA1 domain of COLVII; IgG against LAM5 and LAM6	IB	–

*Ab, antibodies; BSLE, bullous systematic lupus erythematosus; COLVII, collagen VII; F, female; IB, immunoblotting; LAM, laminin; M, male; Pt, patient; SLE, systematic lupus erythematosus; WB, Western blot*.

It has also been reported that EBA is associated with inflammatory bowel disease (IBD) in up to 50% of patients (151). Indeed, a high prevalence of circulating antibodies against Coll VII in IBD patients has been detected (151, 152). Furthermore, Lohi et al. evidenced the presence of Coll VII in the intestinal epithelium (153). Therefore, it has been speculated that autoimmunity to Coll VII, which is present in both gut and skin, might justify the frequent association between EBA and IBD. Moreover, Coll VII antibodies in Crohn’s disease patients may be an ES phenomenon. Veritably, inflammation provoked by Crohn’s disease may alter the intestinal epithelial BMZ, leading to an ongoing autoimmunity to Coll VII (154).

A few cases of psoriasis associated with EBA have been reported (155–161). In these cases, the role of ES is not clear and it could be postulated that its involvement is due to the long-lasting psoriasis lesions. Specifically, a damage to the BMZ could induce circulating non-pathogenic autoantibodies that lead to development of immunobullous diseases (161).

Epitope spreading phenomenon has been thought also as the leading cause of the concomitant presence of two different autoimmune bullous skin diseases in the same patient (162–167) (Table 3). As reported by Yang et al. ES phenomenon could be the explanation of a case of typical pemphigoid gestationis with IgG antibodies to Coll VII (167). In this report, the patient showed a rash on the trunk and limbs with intense erythema, vesicles, and bullae one month postpartum. IB detected IgG autoantibodies reacting against BP230 in the epidermal extracts and 290 kDa Coll VII in the dermal extracts; BP180 antibodies were detected only using an ELISA based on BP180 antigen. Therefore, the authors concluded that these immunopathological findings could be explained by intermolecular ES phenomenon (167). Jonkman et al. described recently a female patient with inflammatory EBA showing non-scarring oral and vaginal involvement (163). Circulating IgG autoantibodies to Coll VII and α3 chain of laminin 332 were simultaneously detected by IB. To further confirm the simultaneous presence of autoantibodies against laminin 332 and Coll VII, IIF on skin substrates lacking BMZ molecules was performed. IgG bound to the BMZ of specimens lacking laminin 332 and Coll VII, revealing the simultaneous presence of two autoantibodies. Therefore, a simultaneous diagnosis of EBA and anti-laminin 332 MMP was made. The significance of the anti-laminin 332 antibodies remains obscure, but it may be determined by the pathogenic presence of anti-Coll VII autoantibodies; indeed, cryptic laminin 332 epitopes could be uncovered in lesional skin, leading to intermolecular ES. The fact that laminin 332 and Coll VII are intimately connected, because the β3 chain of the former binds the NC1 domain of the latter, could suggest a mechanism dependent of physical association of antigens.

**Table 3:** EBA associated with other autoimmune bullous disease.

Reference	Number of patients	Age and sex	Other disease	Laboratory findings	Method
Kawachi et al. (162)	1	1, M	BP	IgG against BP180 NC16A domain	IB
Jonkman et al. (163)	1	64, F	MMP	IgG against laminin α3 subunit	IB
Furukawa et al. (164)	1	29, F	Anti-p200 pemphigoid	IgG against γ1 subunit	IB
Buijsrogge et al. (165)	1	70, M	EBA	IgA against plectin	IB
Osawa et al. (166)	1	75, M	LABD	IgG against 120 kDa antigen	IB
Yang et al. (167)	1	22, F	PG	IgG against BP180 and BP230	IB

*BP, bullous pemphigoid; EBA, epidermolysis bullosa acquisita; F, female; IB, immunoblotting; LABD, linear IgA bullous dermatosis; M, male; PG, pemphigoid gestationis*.

### Linear IgA Bullous Dermatosis (LABD)

Linear IgA bullous dermatosis is an autoimmune subepidermal bullous disease that may be idiopathic or drug-induced (168). Pathologically, LABD is characterized by linear deposition of IgA at the BMZ (168). Dermal and epidermal antigens are identified by IgA, IgG, and by both antibody isotypes (169, 170). Proteolytic cleavage product of BP180 are the major antigen, but also BP230 and Coll VII are less commonly detected (169, 170). In the epidermis, BP180 is the major and possibly initiating antigen (169, 170). Indeed, different binding sites for the LABD antibodies have been reported on BP180 in studies in which the full-length protein, the 120-/97-kDa fragments of its soluble ectodomain and the immunodominant NC16A domain were employed (169–175). These studies showed that intramolecular ES could occur within BP180 leading to the simultaneous binding of IgA antibodies to intact BP180 and its soluble ectodomain. Allen et al. reported in a recent study that no sera identified the dermal 97-kDa protein, while they detected antibody response to a 285 kDa antigen (LABD285) and BP180, suggesting that intermolecular ES of the antigens of the extracellular matrix/dermal components of the basement membrane plays a role in LABD etiopathology (169). In another study, Allen et al. reported that 35% of LABD sera targeted single antigens and 42% targeted two or more antigens with IgA antibodies (170). The presence of multiple epidermal antigens was commoner in adults (51%) than in children (25%), and might be due to a continual antigenic stimulation. Therefore, they suggested that both intra- and intermolecular ES led to the multiplicity of IgA target antigens (170). Indeed, they reported that 34 of 72 IgA antibodies binding BP180 also bound BP230. In addition, they showed that of 72 LABD sera binding BP180, 23 bound also both BP230 and LABD285, and two bound LABD285. Intramolecular ES within BP180 was reported more rarely, with 19 of 72 sera also binding the 97-kDa soluble ectodomain. In contrast to IgA, intermolecular ES with IgG antibodies was rarely reported, and did not involve LABD285. In conclusion, Allen et al. have proposed that the intermolecular ES is frequent in LABD, is age related, and is associated with IgA antibodies rather than IgG antibodies (170). Recently, Sakaguchi et al. described three patients, in whom ELISA detected IgG and IgA antibodies to various subunits of laminin 332, in addition to IgG and IgA reactivity to Coll VII, γ1 subunit, and BP230 and BP180 recombinant proteins (176). A strong IgG and IgA reactivity to laminin 332 has been seldom described in the literature. The authors postulated that the production of autoantibodies against different BMZ antigens was led by ES. Even if the original epitopes in these cases were not detectable, the first immune response might be directed to laminin 332 because of the marked and constant reactivity. Nevertheless, because of the variegated antigen subset in LABD, several patients with peculiar laboratory findings whose epitope/antigen pattern could be due to ES have been described in the literature (177–199) (Table 4).

**Table 4:** Characteristics of LABD patients with peculiar laboratory findings.

Reference	Number of patients	Findings	Method	Note
Kanitakis et al. (177)	1	IgA against BP230	WB	–

Zambruno et al. (178)	1	IgA against 290 kDa antigen of anchoring fibrils	WB	40 years old, M

Berard et al. (179)	1	IgA against BP230	WB	18 months old, F

Hashimoto et al. (180)	1	IgA against CollVII	IB	–

Kawahara et al. (181)	2	IgG and IgA antibodies on dermal side of SSS	IIF	–

Ghohestani et al. (182)	6	IgA against BP230	IB	–
	5	IgA against BP180		

Honoki et al. (183)	1	IgG against a 230 kDa epidermal antigen	IB	54 years old, F

Wakelin et al. (184)	1	IgA against CollVII	WB	76 years old, M

Nie et al. (185)	14	IgA against the XV collagenous domain of BP180, and NC16A of BP180	IB	–

Lin et al. (186)	4	IgA against NC16A domain of BP180	IB	–

Metz et al. (187)	1	IgA and IgG against BP180	WB	39 years old, F

Shimizu et al. (188)	1	Linear deposition of IgA and IgG along the BMZ	DIF	78 years old, M; localized IgA/IgG LABD

Passos et al. (189)	1	Linear deposition of IgA and IgG along the BMZ	DIF	21 years old, F

Yanagihara et al. (190)	1	Linear deposition of IgA and IgG along the BMZ	DIF	35 years old, M; Vogt-Koyanagi-Harada disease

Sakaguchi et al. (176)	3	Pt 1 IgG against laminin α3, laminin β3, laminin γ2; IgA against laminin α3, laminin β3, laminin γ2, Coll VII, Laminin γ1, BP180-C	IB	81 years old, F; K pancreas
		Pt 2 IgG against laminin α3, laminin γ2; IgA against lamininα3, laminin γ2, BP230, BP180-C		88 years old, M; K colon
		Pt 3 IgG against laminin γ2, BP180-N; IgA against laminin α3,		64 years old, M

Kern et al. (191)	1	IgA against the 120 kDa ectodomain of BP180 and Dsg3		48 years old, M; Ulcerative colitis; Overlap LABD and IgA Pemphigus?

Tashima et al. (192)	1	IgA against BP180 NC16A	IB	84 years old, M

Zenke et al. (193)	1	IgA against the 145- and 165-kDa α3 subunits of laminin 332	IB	62 years old, M

Izaki et al. (194)	1	IgA and IgG against the 165-kDa and 145-kDa forms of α3 subunit and the 105-kDa γ2 subunit of laminin 332	IB	53 years old, M

Li et al. (195)	1	IgG and IgA against laminin α 3, laminin-β3 and laminin γ2; IgG and IgA against α6 and β4 subunit of integrin	IB	80 years old, M

Fernandes et al. (196)	1	Linear deposition of IgA and IgG along the BMZ	DIF	7 years old, M

Izaki et al. (197)	1	Linear deposition of IgA and IgG along the BMZ	DIF	5 months old, M

Koga et al. (198)	1	IgG against desmocollin 1	ELISA	70 years old, M; transition from LABD to PH/coexistence of both LABD and PH

Matsuura et al. (199)	1	IgA against NC16A domain of BP180; no reaction to LAD1 shed ectodomains of BP180	IB	29 years old, F; pregnancy (38th week)

*BMZ, basal membrane zone; BP, bullous pemphigoid; K, carcinoma; COLL, collagen; DIF, direct immunofluorescence; Dsg, desmoglein; F, female; IB, immunoblotting; IIF, indirect immunofluorescence; M, male; PT, patient; PH, pemphigus herpetiformis; SSS, salt-split skin; WB, Western blot*.

The ES phenomenon has been also proposed to be involved in LABD patients with psoriasis (200, 201). Cooke et al. reported the case of a 29-year-old man with plaque psoriasis who developed LABD after herpes zoster infection. IB detected IgA antibodies against BP180 and BP230 antigens, and IgG autoantibodies that reacted weakly to the BP180 antigen (200). A similar case was previously reported by Takagi et al. who described the simultaneous presence of psoriasis and LABD in a 68-year-old man after an active hepatitis C infection. In this case, IB detected IgA antibodies against a 97-kDa target antigen in epidermal extracts (201).

LABD has been also reported in association with autoimmune rheumatic diseases, including dermatomyositis, rheumatoid arthritis, and Sjögren’s syndrome (202–206). However, the role of ES in these associations is under debate.

The association between LABD and ulcerative colitis (UC) has been widely reported. Indeed, Paige et al. reported that 8 out 70 LABD patients were also affected by UC (207). In all cases, UC preceded LABD onset by a median of 6.5 years. In addition, a few single cases of LABD associated with UC have been reported (191, 207–211) (Table 5). The reason for this association is unclear, but alteration in colonic mucosal B cell and IgA production may play a pivotal role in LABD onset.

**Table 5:** LABD patients with UC.

Reference	Number of patients	Sex	Age at UC diagnosis	Age at LABD diagnosis
Chan et al. (208)	1	M	76	80

Paige et al. (207)	8	M	12	20
		M	36	41
		M	52	60
		F	30	38
		M	59	60
		F	21	64
		F	7	8
		M	45	47

De Simone et al. (209)	1	–	–	–

Chi et al. (210)	1	F	11	41

Keller et al. (211)	1	M	54	54

Kern et al. (191)	1	M	-	48

*F, female; LABD, linear IgA bullous disease; M, male; UC, ulcerative colitis*.

### Dermatitis Herpetiformis

In gluten sensitive individuals, gluten can lead to pathological immune responses against both gluten components and transglutaminases (TGs). DH belongs to gluten-induced autoimmune diseases. DH is characterized by chronic, pruritic, polymorphic, papulovesicular, or rarely blistering lesions. Histologically DH, such as other subepidermal autoimmune diseases, presents subepidermal blisters neutrophil and eosinophil infiltration and granular IgA deposition in the dermal papilla by DIF. DH patients show circulating IgA to TG3, which is the pivotal autoantigen (212, 213). In most of DH patients, IgA to TG2 are detected, indicating an underlying, usually latent or mild celiac disease (CD) (212, 213). A TG6 autoimmunity may also be detected in some DH patients (212, 213). Furthermore, in a few DH patients, no circulating IgA to TG3 have been reported, but only circulating TG3-IgA immune complexes (212, 213). All three enzymes (TG2, TG3, and TG6) bind deamidate gliadin or other gluten peptides. This diversification of autoimmune antigens could be explained by intermolecular ES. Indeed, it has been postulated that a continued exposure to gliadin could lead to development of IgA anti-TG3 antibodies in patients who already have IgA anti-TG2 antibodies; a subgroup of those who develop IgA anti-TG3 antibodies then develop DH (214). The presence of IgA anti-TG2 antibodies in most DH patients and a higher prevalence of IgA anti-TG3 antibodies in adults than in children with CD are two of the main findings in support of the ES phenomenon (213). In fact, it has been thought that ES could be an explanation for the DH late onset in comparison to CD, which often involves pediatric patients. In this regard, it has been hypothesized that ES and/or cross-reactivity between TG2 and TG3 could lead to TG3 autoimmunity, leading to circulating IgA-TG3 immune complexes formation, which determines skin features in some CD patients (213). However, the possible mechanism of a directly gluten-induced TG3 autoimmunity could not be excluded (213).

Several cases of patients with a history of DH and BP have been reported (215–228) (Table 6). Van der Meer was the first to describe a patient showing clinically both DH and BP features, but only pathological and DIF BP features were found (215). In six cases, DH preceded BP by 4 months to 11 years (223–225). Ameen et al. reported a case of DH that evolved into BP 11 years after its original diagnosis (225). Initially, DIF showed linear BMZ staining with IgG and C3, associated with intensefibrillar IgA staining in the dermal papillae. IIF detected circulating anti-BMZ IgG with epidermal binding on SSS. After 11 years, the response to dapsone and gluten-free diet failed, and a new skin biopsy was performed, revealing overlapping BP/DH immune-histochemical features. Therefore, the authors concluded that the evolution from DH into BP was due to intermolecular ES phenomenon, postulating that neutrophilic infiltrate of DH caused BMZ disruption, leading to the exposure of BP antigens to autoreactive lymphocytes (155, 225). In 11 cases, dual findings for BP and DH were detected by DIF (220–227). On the one hand, BP and DH were described as concomitant in nine cases (220, 221, 223–227); on the other hand, in two patients, DIF was performed at different time, showing only one disease per biopsy (222, 228). In five of the nine patients, serum antibodies were directed against the BMZ, and in one patient only antiendomysial antibodies were highlighted at the DH onset (223). Only two patients with serum autoantibodies compatible with both BP and DH have been described, however, sera were not taken at the same time (222, 228). Indeed, only Schulze et al. detected both BP and DH serum autoantibodies at the same time (227). In addition, Vaira et al. analyzed the HLA profile in a patient, who developed DH several years after the BP diagnosis. HLA alleles were HLA-DQB1 03:01 (predisposing to BP), HLA-DQA1 05:05/05:01 (predisposing to CD) and HLA-DQB1 02:01 (predisposing to CD and to DH) (228). The authors concluded that those findings highlighted a specific genetic susceptibility to both diseases in the same patient (228).

**Table 6:** Patients with a history of DH and BP.

Reference	Number of patient	Clinic	Pathology	DIF positive for both BP and DH	Note
Van der Meer et al. (215)	1	DH + BP	BP	–	BP features detected by DIF
Honeyman et al. (216)	1	Switch from DH to BP	Switch from DH to BP	–	–
Jablonska et al. (217)	9	Concomitant DH and BP	Concomitant DH and BP	–	–
Bean et al. (218)	7	DH	Concomitant DH and BP	–	–
Honeyman et al. (219)	1	Concomitant DH and BP	Concomitant DH and BP	–	Initially negative, later circulating BP antibodies
Jolliffe et al. (220)	1	Concomitant DH and BP	BP	+	57 years old, F
De Jong et al. (221)	1	Concomitant DH and BP	Concomitant DH and BP	+	–
Jawitz et al. (222)	1	DH	BP; only DH 5 years later	+	41 years old, F
Sander et al. (223)	1	First DH; BP after 5 years	First DH; BP after 5 years	+	68 years old, M
Setterfield et al. (224)	1	First DH; both DH and BP 25 years after	First DH; both DH and BP 25 years after	+	58 years old, M
Ameen et al. (225)	1	First DH; BP 11 years after	First DH; both DH and BP 11 years after	+	84 years old, F
Murphy et al. (226)	3	First DH; DH and BP later (4 months, 1 and 11 years)	-	+	83 years old, 2 M; 84 years old, F
Schultze et al. (227)	1	DH	Non-specific for BP or DH	+	77 years old, M; BP and DH serum autoantibodies simultaneously
Vaira et al. (228)	1	First BP; later DH	DH during the follow-up	+	48 years old, M

*BP, bullous pemphigoid; DH, dermatitis herpetiformis; DIF, direct immunofluorescence; F, female; M, male*.

### Mucous Membrane Pemphigoid

Mucous membrane pemphigoid includes different autoimmune subepithelial blistering diseases mainly involving mucous membranes, including the mouth, ocular mucosae, and mucous membranes of the nose (229). Several epithelial basement membrane components have been reported as potential targets of MMP, including BP180, BP230, Coll VII, laminin 332, laminin 311, α6 and β4 integrin subunits (229). The main MMP features is the presence of linear deposits of immunoglobulins (IgG and/or IgA), and/or complement fragments at the dermal–epidermal and/or chorioepithelial BMZ (230). Due to low circulating autoantibodies titers IIF is often negative. For diagnosis detection by ELISA or IB of two major targets of MMP BP180, principally its extracellular domain (231–233), and laminin 332, which are both constitutive elements of anchoring filaments can be of confirmatory value. Therefore, MMP is divided into two major types, anti-BP180-MMP and anti-laminin332-type MMP (234).

The involvement of ES phenomenon in MMP has been postulated in several studies (235–238). It has been thought that in BP180-MMP the development of autoantibodies to laminin 332, which has a pathogenic role in the disease (239), is produced *via* ES from BP180 C terminal domain to laminin 332, because of physical interaction of BP180 C terminal domain and laminin 332 in epidermal BMZ (238). Indeed, Yasukochi et al. reported that IgG or IgA antibodies against various subunits of laminin 332 were detected in 54 of 332 cases of BP180-MMP (238). Bernard et al. presumed the involvement of ES phenomenon in a recent study on laminin 332-MMP, which has reported to be associated with a raised relative risk for neoplastic diseases (235). On the one hand, anti-BP180 auto-antibodies have been detected in up to 75% MMP patients (240); on the other hand, autoantibodies against BP230 have only been occasionally identified in MMP serum, as reported also by Bernard et al. (235). Furthermore, anti-BP230 antibodies have been more oftenly detected in anti-laminin 332 MMP patients (235). This association has been described as determined by ES phenomenon. In point of fact, it has been postulated that damage to BMZ might lead to an abnormal exposure of intracellular antigens such as BP230. In addition, Bernard et al. concluded that the phenomenon of ES could be also postulated for the NC16A domain of BP180, which is strongly immunogenic (231, 235). Another interesting case of possible intermolecular ES was reported by Inoue et al. (104). The authors described one MMP patient, who showed only anti-BP230 autoantibodies. Indeed, IgG antibodies reacted neither with the BP180 NC16A domain nor with the C-terminal domain. Moreover, IB and ELISA identified exclusively anti-BP230 antibodies in the patient’s serum. Furthermore, patient serum did not show reactivity either with any subunits of laminin 332 or with the 120-kDa fragments of BP180 (LAD1). Since the release of autoantibodies to the cytoplasmic protein BP230 is considered a secondary phenomenon, the authors speculated that intermolecular ES could occur because of the oral mucosa ulcerations that led to IgG anti-BP230 production (241). In this context, Hayashi et al. described a MMP patient with blisters who showed IgA and IgG autoantibodies that targeted both laminin 332 and BP180 (242).

The ES phenomenon may be also involved in MMP patients with a history of different autoimmune diseases that involve skin, mucous membrane or different organs (243–251) (Table 7). In a recent study on 6 MMP patients, Zakka et al. postulated the involvement of ES phenomenon in patient with both MMP and mixed connective tissue disease (MCTD) (244). MCTD is a systemic autoimmune disease with mixed features of SLE, dermatomyositis, rheumatoid arthritis, scleroderma, and polymyositis (252). The target antigen is a complex of small nuclear ribonucleoproteins (snRNPs), including a 70 kDa polypeptide (snRNP70) (252). The authors observed that all the 6 MMP patients, which showed antibodies against BP180, BP230, and subunit of human β4 integrin, showed antibodies also to snRNP70 (244). Moreover, they reported that all MMP patients carried either the HLA II alleles DQb1*0301, *0302, or *0603 that have been described as associated with MMP (244). However, only three of six patients carried the HLA alleles associated with MCTD (HLADR4). Although the remaining three patients did not have any allele associated with MCTD, they had MCTD clinical features and serological markers (244). The presence of one autoimmune disease could induce tissue damage and exposure to the immune system of a previously masked epitope. Therefore, the authors concluded that it was possible for the patients who lacked the HLA II genes related to MCTD to produce autoantibodies to snRNP70 as result of an ES phenomenon. In addition, Malik et al. described 8 patients who showed an association of MMP with SLE, MCTD, or both (243). Among them, four patients developed MMP and SLE or MCTD simultaneously, while the other four patients developed SLE, MCTD or both after the MMP diagnosis in a period ranging from 2 to 10 years. ES phenomenon could be also involved in the association between subepidermal autoimmune blistering diseases and IBD, as postulated by Shipman et al. (245). In a recent article, the authors reviewed the literature about this association, finding out 48 cases of IBD patients who showed also a subepidermal blistering disease. More specifically, they reported two female patients who developed MMP several years after the first IBD diagnosis. In both cases, DIF showed linear staining for IgG and C3 at the BMZ. However, only in one case IB detected IgG antibodies to the BP180 antigen. Therefore, the authors theorized that exposure to antigens in the gastro-intestinal tract eventually led to cutaneous disease, which was also supported by the positive DIF in the colon in two LAD patients (253) (Table 7). A supposed intermolecular ES was reported by Ohata et al., who described a singular patient who was affected simultaneously by pemphigus herpetiformis (PH) and MMP with autoantibodies to the laminin γ2 subunit, Dsc1, and BP180 C-terminus (249). Therefore, the authors postulated that ES from Dsc1 to BMZ antigens might lead to the simultaneous occurrence of PH and MMP, although such phenomenon was not demonstrated (249). Kaune et al. described an 87-year old Caucasian female patient with complete symblepharon and dysphagia who showed by IB IgG4 autoantibodies both to the 200 kDa antigen and to the C-terminus of laminin γ1 subunit; in addition, IgA autoantibodies to the C-terminal fragment of BP180 4575-antigens and IgG autoantibodies to BP180 NC16A have been detected in the same patient by IB (250). The authors postulated the involvement of ES, describing the coexistence of p200/laminin γ1 subunit-pemphigoid and MMP. Monshi et al. reported the case of a Caucasian woman affected by anti-p200 pemphigoid who developed antiepiligrin cicatricial pemphigoid, a rare and major subtype of MMP, 17 months after the first diagnosis (247). However, this patient never developed mucosal lesions. In these cases, the ES phenomenon could be postulated but not demonstrated as the cause of two concomitant diseases.

**Table 7:** MMP patients associated with other autoimmune diseases.

Reference	Number of patients and disease	Multiple antigens	Method	Note
Malik et al. (243)	2 concomitant MMP + SLE	Ab against β4 integrin subunit (8 Pt); Ab against BP180 (6 Pt); Ab against BP180 and a 240 kDa antigen (2 Pt)	IB	Coexistence of MMP, SLE and/or MCTD
	2 concomitant MMP + MCTD			
	2 MMP who developed SLE			
	1 MMP who developed MCTD			
	1 MMP who developed SLE/MCTD			

Zakka et al. (244)	3 MMP	Ab against snRNP70	ELISA	Pt who lacked any HLA II genes associated with MCTD may produce *via* epitope spreading Ab against snRNP70

Shipman et al. (245)	1 UC; 1 CD	Linear staining for IgG and C3 at the BMZ	DIF	IB showed IgG against BP180 only in Pt with Crohn’s disease
		IgG anti-BMZ on the dermal side of SSS	IIF	

Takegami et al. (246)	1 concomitant MMP + LAD + SS	IgG and IgA against laminin α3 subunit; IgA against 120 kDa antigen	IB	Simultaneous diagnosis of MMP, LAD, and SS

Monshi et al. (247)	1 anti-p200/anti-LAM γ1 pemphigoid	IgG against α3 chain of laminin 332	IB	86 years old, F; no mucosal involvement

Yamada et al. (248)	1 PNP	IgG against γ2 subunit of laminin 332	IB	68 years old, M; K thyroid, kidney CCK, follicular dendritic cell sarcoma in the retroperitoneal area.

Ohata et al. (249)	1 PH	IgG and IgA against BP180 C-terminus; IgG against γ2 subunit of laminin 332	IB	63 years old, M; no mucosal involvement

Li et al. (250)	1 anti-p200/anti-LAM γ1 pemphigoid	IgG against γ1, α3 and β3 subunit of laminin 332	IB	72 years old, M; psoriasis

Kaune et al. (251)	1 anti-p200/anti-LAM γ1 pemphigoid	IgA against C-terminal fragment of BP180; IgG against BP180 NC16A	IB	87 years old, F

*Ab, antibody; BMZ, basal membrane zone; CD, Crohn’s disease; DIF, direct immunofluorescence; F, female; M, male; IIF, indirect immunofluorescence; IB, immunoblotting; LAD, linear IgA dermatosis; MCTD, mixed connective tissue disease; MMP, mucous membrane pemphigoid; Pt, patient; SLE, systemic lupus erythematosus; SS, Sjögren syndrome; UC, ulcerative colitis; BP, bullous pemphigoid; K, carcinoma; CCK, clear cell carcinoma; PH, pemphigus herpetiformis; PNP, paraneoplastic pemphigus*.

On the contrary, when the transition from one disease to another is accompanied by shifting of reactivity from one antigen to another, the ES phenomenon is strongly probable. Several cases of clinical switch from a skin disease to MMP have been reported in the literature (60, 254–257) (Table 8). Chan et al. reported in 1991 five Steven-Johnson’s syndrome (SJS) patients, who developed MMP (254). SJS is a life-threatening dermatosis with high morbidity and mortality. These patients developed MMP, characterized by chronic ocular mucosal scarring lesions. The linear immune deposits along the oral or ocular mucosal BMZ in all five patients fulfilled the diagnostic criteria for MMP. In addition, immunoblot analysis of the serum of two of five patients identified a 120-kDa epidermal antigen, which may represent one of the MMP autoantigens (258). The authors concluded that the mucosal injury occurred in SJS resulted in ES phenomenon involving the 120-kDa epithelial antigen (254). Similarly, Fania et al. described two male patients who developed MMP with chronic ocular mucosal scarring lesions after TEN (257). In both patients, DIF on perilesional conjunctiva showed IgG and C3 deposits along the BMZ in a linear pattern, while IIF and ELISA for BP180 and BP230 were negative. Although the association between TEN and MMP may be coincidental, the authors hypothesized that chronic eye surface injury might represent an immunologic trigger, leading to the ES phenomenon. Moreover, five cases similar to those reported by Fania et al. were described by De Rojas et al. (255) (Table 8).

**Table 8:** Reported case of switch to MMP.

Reference	Number of patients and disease	DIF (number of patients)	IIF (number of patients)	ELISA (number of patients)	IB (number of patients)	Note	Clinical switch
Chan et al. (254)	5 SJS	Performed (5/5)	Performed (2/5)	NP	Pt 1 120 and 140 kDa epidermal antigen	Pt 1 59 years old, M; MMP onset 6 months after SJS	From SJS to MMP
						Pt 2 6 years old, M; MMP onset 31 years after SJS	
					Pt 2 120 kDa epidermal antigen	Pt 3 22 years old, M; MMP onset 14 months after SJS	
						Pt 4 33 years old, F; MMP onset 2 years after SJS	
						Pt 5 34 years old, F; MMP onset 2 years after SJS	

De Rojas et al. (255)	5 LS	Performed (1/5)	NP	NP	NP	–	From LS to MMP

Mignogna et al. (256)	2 OLP	Performed (2/2)	Performed (2/2)	Ab against BP180	NP	Pt 1 72 years old, F	From OLP to MMP
						Pt 2 64 years old, F	

Fania et al. (257)	2 LS	Performed (2/2)	Performed (2/2)	Performed (2/2)	NP	Pt 1 80 years old, M; MMP onset 6 months after LS	From LS to MMP
						Pt 2 60 years old, M; MMP onset 2 months after LS	

Sardy et al. (60)	BP	Performed	NP	IgG against laminin 332	IgG4 against laminin 332	18 years old, F; MMP transition 5 years after the BP diagnosis	From BP to MMP

*BP, bullous pemphigoid; DIF, direct immunofluorescence; F, female; IIF, indirect immunofluorescence; IB, immunoblotting; LS, Lyell’s syndrome; M, male; MMP, mucous membrane pemphigoid; NP, not performed; OLP, oral lichen planus; Pt, patient; SJS, Steven-Johnson’s syndrome*.

Finally, it has been also reported that MMP can arise in patient with hematological disease or solid malignancies, possibly involving the ES phenomenon (259–267). It has been postulated that the massive alteration of tumoral cells or tissues could lead to an autoimmune disease through cross-reactive antigens or exposure of criptic epitopes followed by an ES phenomenon.

### Lichen Planus Pemphigoides

Lichen planus pemphigoides is a rare autoimmune blistering disease that occurs in association with LP. DIF shows linear deposits of IgG and C3 along the BMZ with fibrillary deposits of fibrin at the epithelial/lamina propria junction. By IB and ELISA LPP sera are strongly reactive with BP180. More specifically, LPP sera reacted with AA 46–59 of NC16A domain, previously shown to be unreactive with BP sera (268). Mignogna et al. reported two cases of oral lichen planus (OLP) who switched to MMP in a period ranging from 3 to 11 years (256). They hypothesized that the patients might be an example of the ES phenomenon suggesting that LPP might not exist as a separate disease, but might be the result of the ES from one disease (OLP) to another (BP/MMP), where the lichenoid papules arise before the vesciculobullous lesions or vice versa. In particular, the BMZ break, due to mast cell degranulation, might have continuously exposed BMZ proteins, such as BP180 and laminin-322, that may lead to an autoimmune humoral response (256). Another case from Sekiya et al. presented at first LP lesions on the hands, and than developed blisters on her limbs and erosions of the buccal mucosa (269). The patient’s serum reacted to IgG autoantibodies against the BP180 NC16A domain, the BP180 C-terminal domain and Dsg1. However, a serum sampled one and a half years before the diagnosis of LP did not shown activity to BP180 domains. These findings may evidence that the damage to the basal cells in LP exposed a sequestered antigen or formed neoantigens, producing pathogenic autoantibodies that lead to LPP. Most of the previous LPP cases showed autoantibodies to the NC16A domain of BP180. This report demonstrates the presence of circulating autoantibodies to BP180 only after the development of blisters, suggesting the role of sequestered antigen exposure or neoantigens in the LPP etiopathogenesis.

## Implications for Therapy

Treatment regimens for autoimmune blistering skin diseases rely on general immunosuppression, typically with corticosteroids, steroid-sparing immunosuppressive drugs, and/or adjunctive treatments such as intravenous immunoglobulins or plasmapheresis. The B cell-depleting anti-CD20 antibody (rituximab) has emerged as a major option for pemphigus patients with steroid-resistant disease or when there are adverse effects with conventional therapies. Data from a recent clinical trial suggest that first-line use of rituximab plus short-term prednisone is safe and more effective than using prednisone alone (270). However, these approaches impair the efficiency of the immune response and render the treated subjects to be more susceptible to the infections. In addition, since patient morbidity and even mortality results from side effects of treatment, the development of more targeted therapies that leave the immune system functional and able to neutralize pathogens is desirable.

To overcome this problems therapy for autoimmune diseases should eliminate only pathogenic autoimmune cells such as performed by Ellebrecht et al. for pemphigus therapy. They have recently demonstrated that autoantigen-based chimeric immunoreceptors can direct T cells to kill Dsg3-specific B cells *in vivo* (271). Another target specific approach could be based on therapeutical peptides (such as decoy peptides) able to specifically deplete pathogenic autoantibodies (45). The association of PV with human leukocyte antigen class II alleles has highlighted the role of the presentation of immunodominant peptides to autoreactive T helper cells. In parallel, the correlation of disease activity and autoantibodies of the IgG4 and IgE subclasses underlines the role of T helper 2 cells as critical regulators of pemphigus pathogenesis. In this context, novel therapeutic approaches that target autoreactive T cells functions could be also relevant in future. In this context, therapeutic peptide vaccine approach for the restoration of immune tolerance is currently investigated (272, 273). An interesting study on MS, which has led to a phase I clinical trial in humans, has used peripheral blood mononuclear cells chemically engineered to present peptides (274). Thus, the evidence that an ES phenomenon may change the epitope/antigen profile during the course of the disease and the demonstration of its pathological role could make the development of epitope/antigen-specific therapies more difficult.

An ideal therapeutic approach should be able to eliminate not only specific pathogenic autoantibodies present at disease onset but also those arising during the course of disease through the ES phenomenon. In addition, it should be considered that whereas the epitope/antigen specific therapeutic approaches involve untreated bullous disease patients, the performed studies involving treated patients could have underestimated the impact of ES in disease progression.

In the light of published data on ES in the autoimmune blistering disease, the possible therapeutic use of decoy peptides to block autoantibody binding *in vivo* may require early and aggressive administration of several different peptides. For example, as for BP, other pathogenic regions of BP180, in addition to NC16A, should be considered (22). On the other hand, in pemphigus disease, the hypothesis that multiple antigens could be sequentially involved by ES in the pathogenesis of disease (85) could suggest an antigen specific approach based on multiple antigens and not only on the major one. On the other hand, the static nature of the Dsg3-reactive B cell repertoire that persists over time and causes disease relapse in PV patients suggests that Dsg3 ectodomain could be an effective target for PV therapy (20, 271, 275–278). In addition, although autoimmune response may spread to new epitopes during the course of disease, specific epitope/antigen therapies show considerable efficacy in epitope-induced mouse models of autoimmunity (272, 273, 279, 280) suggesting that epitope-specific therapies could also operate at the level of regulating mechanisms of immune tolerance rather than depending only on the interference with specific autoaggressive B and T cells.

## Concluding Remarks

To get insights into the dynamics and role of sequential immune responses to newly arising epitopes/antigens is crucial to understand autoimmune disease pathogenesis. Moreover, this knowledge could be used to design antigen-specific therapies.

Although definition of hierarchy and functional significance of ES in human disease is difficult, this knowledge in animal models of autoimmune disease has been partially obtained. The difficulty to investigate ES in patients is probably due to the immunosuppressive treatments that could impair the diversification of the immune response and the time of ES appearance that, such as occurred in several mouse models, could become detectable before disease onset. However, despite these limitations, in patients affected by autoimmune bullous diseases the functional role of ES has been demonstrated. In particular, in BP patients a significant relation of ES with disease severity at diagnosis has been reported (22). Moreover, data from mouse model and BP patients raise the possibility that the development of autoantibodies against intracellular targets may follow extracellular one and correlates with the initial phase of the disease when the tissue damage starts occurring (22, 58). Although in PV the ES has been rarely found (20), in the clinical transition between mucosal form to the mucocutaneous one seems to have a major role (73, 76). In addition, the growing body of evidences on the existence of other pathogenic autoantibodies targeting non-Dsg antigens located on cell membrane (83, 84) and/or intracellular compartments (85) suggests a role of autoantibody diversification in PV pathogenesis. In parallel, in EPF the role of ES appears to be crucial for the establishment of the autoimmune response (102). Moreover, several reported cases in which a disease transition is preceded by an intermolecular ES phenomenon further underlines the functional role of ES in autoimmune blistering disease.

Autoantigens targeted by ES phenomena in autoimmune blistering disease are often physically linked (for example BP180 and BP230, Laminin 332 and BP180; laminin 332 and Coll VII), Thus, a mechanism dependent on physical association seems to be involved in the disease pathogenesis. In addition, a mechanism independent by physical association, such as tissue damage followed by the exposure of cryptic epitope, at least in BP and PNP, has been postulated.

In conclusion, the definition of hierarchy and functional significance of ES in autoimmune bullous diseases is partially known at present. An important improvement of knowledge on ES phenomenon could be achieved by dissecting the process in active mouse models that are available for the majority of autoimmune blistering diseases (43, 45, 82, 281–285).

## Author Contributions

GZ and DD have designed and written the manuscript.

## Conflict of Interest Statement

The authors declare that the research was conducted in the absence of any commercial or financial relationships that could be construed as a potential conflict of interest.

## Abbreviations

ES, epitope spreading; IIF, indirect immunofluorescence; DIF, direct immunofluorescence; IB, immunoblotting; BMZ, basement membrane zone; Dsg, desmoglein; Coll VII, collagen VII; PV, pemphigus vulgaris; PF, pemphigus foliaceus; EPF, endemic pemphigus foliaceus; MMP, mucous membrane pemphigoid; PNP, paraneoplastic pemphigus; EBA, epidermolysis bullosa acquisita; BP, bullous pemphigoid.

## References

[1] Oldstone MB Molecular mimicry and autoimmune disease. Cell (1987) 51(5):878. 10.1016/0092-8674(87)90507-1 3621346

[2] Gianani R Sarvetnick N. Viruses, cytokines, antigens, and autoimmunity. Proc Natl Acad Sci U S A (1996) 93(6):2257–9. 10.1073/pnas.93.6.2257 8637859 PMC39782

[3] Yang SH Gao CY Li L Chang C Leung PSC Gershwin ME The molecular basis of immune regulation in autoimmunity. Clin Sci (Lond) (2018) 132(1):43–67. 10.1042/CS20171154 29305419

[4] Elkon K Skelly S Parnassa A Moller W Danho W Weissbach H Identification and chemical synthesis of a ribosomal protein antigenic determinant in systemic lupus erythematosus. Proc Natl Acad Sci U S A (1986) 83(19):7419–23. 10.1073/pnas.83.19.7419 2429305 PMC386729

[5] Gordon TP Wolfson-Reichlin M Blalock D Deveshwar S Reichlin M Humoral response in spontaneous and experimental autoimmunity to the ribosomal P proteins. Lupus (1996) 5(4):340–1. 10.1177/096120339600500418 8869910

[6] Kurtzke JF Epidemiologic evidence for multiple sclerosis as an infection. Clin Microbiol Rev (1994) 7(1):141 10.1128/CMR.7.1.141 PMC358295 8269393

[7] Vaughan JH Valbracht JR Nguyen MD Handley HH Smith RS Patrick K Epstein-Barr virus-induced autoimmune responses. I. Immunoglobulin M autoantibodies to proteins mimicking and not mimicking Epstein-Barr virus nuclear antigen-I. J Clin Invest (1995) 95(3):1306–15. 10.1172/JCI117781 7533788 PMC441470

[8] Vaughan JH Nguyen MD Valbracht JR Patrick K Rhodes GH Epstein-Barr virus-induced autoimmune responses. II. Immunoglobulin G autoantibodies to mimicking and nonmimicking epitopes. Presence in autoimmune disease. J Clin Invest (1995) 95(3):1316–27. 10.1172/JCI117782 7533789 PMC441471

[9] Hendrick JP Wolin S Rinke J Lerner MR Steitz JA Ro small cytoplasmic rihonudeoproteins are a subclass of La ribonndeoproteins: further characterization of the Ro and La small ribontxcleop rote ins from uninfected mammahan cells. Mol Cell Biol (1981) 1(12):1138–49. 10.1128/MCB.1.12.1138 6180298 PMC369740

[10] Reichlin M Significance of the Ro antigen system. J Clin Iramtinol (1986) 6(5):339–48. 10.1007/BF00915372 2429977

[11] McRae BL Vanderlugt CL Dal Canto MC Miller SD. Functional evidence for epitope spreading in the relapsing pathology of experimental autoimmune encephalomyelitis. J Exp Med (1995) 182(1):75–85. 10.1084/jem.182.1.75 7540658 PMC2192093

[12] Miller SD Vanderlugt CL Lenschow DJ Pope JG Karandikar NJ Dal Canto MC Blockade of CD28/B7-1 interaction prevents epitope spreading and clinical relapses of murine EAE. Immunity (1995) 3(6):739–45. 10.1016/1074-7613(95)90063-2 8777719

[13] Liebert UG Linington C Ter Meulen V. Induction of autoimmune reactions to myelin basic protein in measles virus encephalitis in Lewis rats. J Neuroimmunol (1988) 17(2):103–18. 10.1016/0165-5728(88)90018-5 2447122 PMC7134202

[14] Liebert UG Hashim GA Ter Meulen V. Characterization of measles virus-induced cellular autoimmune reactions against myelin basic protein in Lewis rats. J Neuroimmunol (1990) 29(1–3):139–47. 10.1016/0165-5728(90)90156-H 1698812 PMC7119477

[15] Liebert UG Ter Meulen V. Synergistic interaction between measles virus infection and myelin basic protein peptide-specific T cells in the induction of experimental allergic encephalomyelitis in Lewis rats. J Neuroimmunol (1993) 46(1–2):217–23. 10.1016/0165-5728(93)90252-T 7689589 PMC7119583

[16] Prasad S Kohm AP McMahon JS Luo X Miller SD. Pathogenesis of NOD diabetes is initiated by reactivity to the insulin B chain 9-23 epitope and involves functional epitope spreading. J Autoimmun (2012) 39(4):347–53. 10.1016/j.jaut.2012.04.005 22647732 PMC3434243

[17] Tuohy VK Yu M Weinstock-Guttman B Kinkel RP. Diversity and plasticity of self recognition during the development of multiple sclerosis. J Clin Invest (1997) 99(7):1682–90. 10.1172/JCI119331 9120012 PMC507988

[18] Goebels N Hofstetter H Schmidt S Brunner C Wekerle H Hohlfeld R. Repertoire dynamics of autoreactive T cells in multiple sclerosis patients and healthy subjects: epitope spreading versus clonal persistence. Brain (2000) 123(Pt 3):508–18. 10.1093/brain/123.3.508 10686174

[19] O’Connor KC Appel H Bregoli L Call ME Catz I Chan JA Antibodies from inflamed central nervous system tissue recognize myelin oligodendrocyte glycoprotein. J Immunol (2005) 175(3):1974–82. 10.4049/jimmunol.175.3.1974 16034142 PMC4515951

[20] Ohyama B Nishifuji K Chan PT Kawaguchi A Yamashita T Ishii N Epitope spreading is rarely found in pemphigus vulgaris by large-scale longitudinal study using desmoglein 2-based swapped molecules. J Invest Dermatol (2012) 132(4):1158–68. 10.1038/jid.2011.448 22277941

[21] Chan PT Ohyama B Nishifuji K Yoshida K Ishii K Hashimoto T Immune response towards the amino-terminus of desmoglein 1 prevails across different activity stages in nonendemic pemphigus foliaceus. Br J Dermatol (2010) 162(6):1242–50. 10.1111/j.1365-2133.2010.09696.x 20163417

[22] Di Zenzo G Thoma-Uszynski S Calabresi V Fontao L Hofmann SC Lacour JP Demonstration of epitope-spreading phenomena in bullous pemphigoid: results of a prospective multicenter study. J Invest Dermatol (2011) 131(11):2271–80. 10.1038/jid.2011.180 21697892

[23] Maeda JY Moura AK Maruta CW Santi CG Prisayanh PS Aoki V. Changes in the autoimmune blistering response: a clinical and immunopathological shift from pemphigus foliaceus to bullous pemphigoid. Clin Exp Dermatol (2006) 31(5):653–5. 10.1111/j.1365-2230.2006.02174.x 16901304

[24] Poole BD Schneider RI Guthridge JM Velte CA Reichlin M Harley JB Early targets of nuclear RNP humoral autoimmunity in human systemic lupus erythematosus. Arthritis Rheum (2009) 60(3):848–59. 10.1002/art.24306 19248110 PMC2653589

[25] Ciubotariu R Liu Z Colovai AI Ho E Itescu S Ravalli S Persistent allopeptide reactivity and epitope spreading in chronic rejection of organ allografts. J Clin Invest (1998) 101(2):398–395. 10.1172/JCI1117 9435312 PMC508579

[26] Liu ZR Colovai AI Tugulea S Reed EF Fisher PE Mancini D Indirect allorecognidon of donor HLA-DR peptides in organ allograft rejection. J Clin Invest (1996) 98:1150–7. 10.1172/JCI118898 8787678 PMC507537

[27] McCluskey J Farris AD Keech CL Purcell AW Rischmueller M Kinoshita G Determinant spreading: lessons from animal models and human disease. Immunol Rev (1998) 164:209–29. 10.1111/j.1600-065X.1998.tb01222.x 9795778

[28] Olson JK Croxford JL Miller SD Virus-induced autoimmunity: potential role of viruses in initiation, perpetuation, and progression of T cell-mediated autoimmune diseases. Viral Immunol (2001) 14(3):227–50. 10.1089/088282401753266756 11572634

[29] Vanderlugt CL Miller SD. Epitope spreading in immune-mediated diseases: implications for immunotherapy. Nat Rev Immunol (2002) 2(2):85–95. 10.1038/nri724 11910899

[30] Poole BD Scofield RH Harley JB James JA Epstein–Barr virus and molecular mimicry in systemic lupus erythematosus. Autoimmunity (2006) 39(1):63–70. 10.1080/08916930500484849 16455583

[31] Cornaby C Gibbons L Mayhew V Sloan CS Welling A Poole BD. B cell epitope spreading: mechanisms and contribution to autoimmune diseases. Immunol Lett (2015) 163(1):56–68. 10.1016/j.imlet.2014.11.001 25445494

[32] Topfer F Gordon T McCluskey J. Intra- and intermolecular spreading of autoimmunity involving the nuclear self-antigens La (SS-B) and Ro (SS-A). Proc Nat Acad Sci U S A (1995) 92(3):875–9. 10.1073/pnas.92.3.875 7846070 PMC42723

[33] Deshmukh US Bagavant H Sim D Pidiyar V Fu SM. A SmD peptideinduces better antibody responses to other proteins within the small nuclearribonucleoprotein complex than to SmD protein via intermolecular epitopespreading. J Immunol (2007) 178(4):2565–71. 10.4049/jimmunol.178.4.2565 17277166

[34] Amigorena S Bonnerot C. Fc receptor signaling and trafficking: a connectionfor antigen processing. Immunol Rev (1999) 172:279–84. 10.1111/j.1600-065X.1999.tb01372.x 10631953

[35] Lake P Mitchison NA Regulatory mechanisms in the immune response tocell-surface antigens. Cold Spring Harbor Symp Quantitat Biol (1977) 41(Pt2):589–95. 10.1101/SQB.1977.041.01.068 268247

[36] Di Noia JM Neuberger MS Molecular mechanisms of antibody somatic hyper-mutation. Annu Rev Biochem (2007) 76:1–22. 10.1146/annurev.biochem.76.061705.090740 17328676

[37] Deshmukh US Bagavant H Lewis J Gaskin F Fu SM. Epitope spreading within lupus-associated ribonucleoprotein antigens. Clin Immunol (2005) 117(2):112–20. 10.1016/j.clim.2005.07.002 16095971

[38] Ray SK Putterman C Diamond B. Pathogenic autoantibodies are routinely generated during the response to foreign antigen: a paradigm for autoimmune disease. Proc Natl Acad Sci U S A (1996) 93(5):2019–24. 10.1073/pnas.93.5.2019 8700878 PMC39902

[39] Satoh M Ajmani AK Stojanov L Langdon JJ Ogasawara T Wang J Autoantibodies that stabilize the molecular interaction of Ku antigen with DNA-dependent protein kinase catalytic subunit. Clin Exp Immunol (1996) 105(3):460–7. 10.1046/j.1365-2249.1996.d01-775.x 8809135 PMC2200543

[40] Simetsek PD Campbell DG Lanzavecchia A Eairweather N Watts C. Modulation of antigen processing by bound antibodies can boost or suppress class II major histocompatibility complex presentation of different T cell determinants. J Exp Med (1995) 181(6):1957–63. 10.1084/jem.181.6.1957 7539034 PMC2192058

[41] Watts C Lanzavecchia A. Suppressive effect of antibody on processing of T cell epitopes. J Exp Med (1993) 178(4):1459–63. 10.1084/jem.178.4.1459 7690836 PMC2191211

[42] Di Zenzo G Della Torre R Zambruno G Borradori L. Bullous pemphigoid: from the clinic to the bench. Clin Dermatol (2012) 30:3–16. 10.1016/j.clindermatol.2011.03.005 22137222

[43] Ujiie H Shibaki A Nishie W Sawamura D Wang G Tateishi Y A novel active mouse model for bullous pemphigoid targeting humanized pathogenic antigen. J Immunol (2010) 184(4):2166–74. 10.4049/jimmunol.0903101 20089696

[44] Zillikens D Rose PA Balding SD Liu Z Olague-Marchan M Diaz LA Tight clustering of extracellular BP180 epitopes recognized by bullous pemphigoid autoantibodies. J Invest Dermatol (1997) 109(4):573–9. 10.1111/1523-1747.ep12337492 9326393

[45] Nishie W Sawamura D Goto M Ito K Shibaki A McMillan JR Humanization of autoantigen. Nat Med (2007) 13(3):378–83. 10.1038/nm1496 17322897

[46] Yamamoto K Inoue N Masuda R Fujimori A Saito T Imajoh-Ohmi S Cloning of hamster type XVII collagen cDNA, and pathogenesis of anti-type XVII collagen antibody and complement in hamster bullous pemphigoid. J Invest Dermatol (2002) 118(3):485–92. 10.1046/j.0022-202x.2001.01683.x 11874488

[47] Liu Z Diaz LA Troy JL Taylor AF Emery DJ Fairley JA A passive transfer model of the organspecific autoimmune disease, bullous pemphigoid, using antibodies generated against the hemidesmosomal antigen, BP180. J Clin Invest (1993) 92(5):2480–8. 10.1172/JCI116856 7693763 PMC288433

[48] Kiss M Husz S Jánossy T Marczinovits I Molnár J Korom I Experimental bullous pemphigoid generated in mice with an antigenic epitope of the human hemidesmosomal protein BP230. J Autoimmun (2005) 24(1):1–10. 10.1016/j.jaut.2004.09.007 15725571

[49] Feldrihan V Licarete E Florea F Cristea V Popescu O Sitaru C IgG antibodies against immunodominant C-terminal epitopes of BP230 do not induce skin blistering in mice. Hum Immunol (2014) 75(4):354–63. 10.1016/j.humimm.2014.01.005 24468586

[50] Murakami H Nishioka S Setterfield J Bhogal BS Black MM Zillikens D Analysis of antigens targeted by circulating IgG and IgA autoantibodies in 50 patients with cicatricial pemphigoid. J Dermatol Sci (1998) 17(1):39–44. 10.1016/S0923-1811(97)00067-4 9651827

[51] Di Zenzo G Grosso F Terracina M Mariotti F De Pità O Owaribe K Characterization of the anti-BP180 autoantibody reactivity profile and epitope mapping in bullous pemphigoid patients. J Invest Dermatol (2004) 122(1):103–10. 10.1046/j.0022-202X.2003.22126.x 14962097

[52] Di Zenzo G Thoma-Uszynski S Fontao L Calabresi V Hofmann SC Hellmark T Multicenter prospective study of the humoral autoimmune response in bullous pemphigoid. Clin Immunol (2008) 128(3):415–26. 10.1016/j.clim.2008.04.012 18571472

[53] Tanaka M Hashimoto T Amagai M Shimizu N Ikeguchi N Tsubata T Characterization of bullous pemphigoid antibodies by use of recombinant bullous pemphigoid antigen proteins. J Invest Dermatol (1991) 97(4):725–8. 10.1111/1523-1747.ep12484223 1940445

[54] Perriard J Jaunin F Favre B Büdinger L Hertl M Saurat JH IgG autoantibodies from bullous pemphigoid (BP) patients bind antigenic sites on both the extracellular and the intracellular domains of the BP antigen 180. J Invest Dermatol (1999) 112(2):141–7. 10.1046/j.1523-1747.1999.00497.x 9989787

[55] Skaria M Jaunin F Hunziker T Riou S Schumann H Bruckner-Tuderman L IgG autoantibodies from bullous pemphigoid patients recognize multiple antigenic reactive sites located predominantly within the B and C subdomains of the COOH-terminus of BP230. J Invest Dermatol (2000) 114(5):998–1004. 10.1046/j.1523-1747.2000.00893.x 10771483

[56] Mariotti F Grosso F Terracina M Ruffelli M Cordiali-Fei P Sera F Development of a novel ELISA system for detection of anti-BP180 IgG and characterization of autoantibody profile in bullous pemphigoid patients. Br J Dermatol (2004) 151(5):1004–10. 10.1111/j.1365-2133.2004.06245.x 15541078

[57] Thoma-Uszynski S Uter W Schwietzke S Schuler G Borradori L Hertl M. Autoreactive T and B cells from bullous pemphigoid (BP) patients recognize epitopes clustered in distinct regions of BP180 and BP230. J Immunol (2006) 176(3):2015–23. 10.4049/jimmunol.176.3.2015 16424234

[58] Di Zenzo G Calabresi V Olasz EB Zambruno G Yancey KB. Sequential intramolecular epitope spreading of humoral responses to human BPAG2 in a transgenic model. J Invest Dermatol (2010) 130(4):1040–7. 10.1038/jid.2009.309 19812601

[59] Olasz EB Roh J Yee CL Arita K Akiyama M Shimizu H Human bullous pemphigoid antigen 2 transgenic skin elicits specific IgG in wildtype mice. J Invest Dermatol (2007) 127(12):2807–17. 10.1038/sj.jid.5700970 17657247 PMC2546607

[60] Sárdy M Borovaya A Horváth ON Folwaczny C Schmitt W Schmidt T Successful rituximab treatment of juvenile bullous pemphigoid with esophageal scarring due to epitope spreading. J Dtsch Dermatol Ges (2016) 14(6):618–21. 10.1111/ddg.12902 27240075

[61] Kasperkiewicz M Hoppe U Zillikens D Schmidt E. Relapse-associated autoantibodies to BP180 in a patient with anti-p200 pemphigoid. Clin Exp Dermatol (2010) 35(6):614–7. 10.1111/j.1365-2230.2009.03731.x 19874345

[62] Ohata C Ishii N Koga H Fukuda S Tateishi C Tsuruta D Coexistence of autoimmune bullous diseases (AIBDs) and psoriasis: a series of 145 cases. J Am Acad Dermatol (2015) 73(1):50–5. 10.1016/j.jaad.2015.03.016 25896671

[63] Mondello MR Magaudda L Pergolizzi S Santoro A Vaccaro M Califano L Behavior of laminin 1 and type IV collagen in uninvolved psoriatic skin. Immunohistochemical study using confocal laser scanning microscopy. Arch Dermatol Res (1996) 288(9):527–31. 10.1007/BF02505249 8874747

[64] Vaccaro M Magaudda L Cutroneo G Trimarchi F Barbuzza O Guarneri F Changes in the distribution of laminin alpha1 chain in psoriatic skin: immunohistochemical study using confocal laser scanning microscopy. Br J Dermatol (2002) 146(3):392–8. 10.1046/j.1365-2133.2002.04637.x 11952538

[65] Chen YJ Wu CY Lin MW Chen TJ Liao KK Chen YC Comorbidity profiles among patients with bullous pemphigoid: a nationwide population-based study. Br J Dermatol (2011) 165(3):593–9. 10.1111/j.1365-2133.2011.10386.x 21517800

[66] Amagai M Klaus-Kovtun V Stanley JR. Autoantibodies against a novel epithelial cadherin in pemphigus vulgaris, a disease of cell adhesion. Cell (1991) 67(5):869–77. 10.1016/0092-8674(91)90360-B 1720352

[67] Ishii K Amagai M Hall RP Hashimoto T Takayanagi A Gamou S Characterization of autoantibodies in pemphigus using antigen-specific enzyme-linked immunosorbent assays with baculovirus-expressed recombinant desmogleins. J Immunol (1997) 159(4):2010–7. 9257868

[68] Ruach M Ohel G Rahav D Samueloff A Pemphigus vulgaris and pregnancy. Obstet Gynecol Surv (1995) 50(10):755–60. 10.1097/00006254-199510000-00023 8524526

[69] Di Zenzo G Amber KT Sayar BS Müller EJ Borradori L. Immune response in pemphigus and beyond: progresses and emerging concepts. Semin Immunopathol (2016) 38(1):57–74. 10.1007/s00281-015-0541-1 26597100

[70] Di Zenzo G Borradori L Muller EJ The pathogenesis of pemphigus: controversy versus complexity. Exp Dermatol (2017) 26(12):1271–3. 10.1111/exd.13176 27571938

[71] Anhalt GJ Labib RS Voorhees JJ Beals TF Diaz LA. Induction of pemphigus in neonatal mice by passive transfer of IgG from patients with the disease. N Engl J Med (1982) 306(20):1189–96. 10.1056/NEJM198205203062001 7040962

[72] Mahoney MG Wang Z Rothenberger K Koch PJ Amagai M Stanley JR. Explanations for the clinical and microscopic localization of lesions in pemphigus foliaceus and vulgaris. J Clin Invest (1999) 103(4):461–8. 10.1172/JCI5252 10021453 PMC408100

[73] Ding X Aoki V Mascaro JM Jr Lopez-Swiderski A Diaz LA Fairley JA. Mucosal and mucocutaneous (generalized) pemphigus vulgaris show distinct autoantibody profiles. J Invest Dermatol (1997) 109(4):592–6. 10.1111/1523-1747.ep12337524 9326396

[74] Harman KE Gratian MJ Bhogal BS Challacombe SJ Black MM. A study of desmoglein 1 autoantibodies in pemphigus vulgaris: racial differences in frequency and the association with a more severe phenotype. Br J Dermatol (2000) 143(2):343–8. 10.1046/j.1365-2133.2000.03660.x 10951143

[75] Amagai M Hashimoto T Green KJ Shimizu N Nishikawa T. Antigen-specific immunoadsorption of pathogenic autoantibodies in pemphigus foliaceus. J Invest Dermatol (1995) 104(6):895–901. 10.1111/1523-1747.ep12606168 7539469

[76] Salato VK Hacker-Foegen MK Lazarova Z Fairley JA Lin MS. Role of intramolecular epitope spreading in pemphigus vulgaris. Clin Immunol (2005) 116(1):54–64. 10.1016/j.clim.2005.03.005 15925832

[77] Ding X Diaz LA Fairley JA Giudice GJ Liu Z. The anti-desmoglein 1 autoantibodies in pemphigus vulgaris sera are pathogenic. J Invest Dermatol (1999) 112(5):739–43. 10.1046/j.1523-1747.1999.00585.x 10233765

[78] Miyagawa S Amagai M Iida T Yamamoto Y Nishikawa T Shirai T Late development of antidesmoglein 1 antibodies in pemphigus vulgaris: correlation with disease progression. Br J Dermatol (2000) 142(4):849. 10.1046/j.1365-2133.1999.03209.x 10606856

[79] Sekiguchi M Futei Y Fujii Y Iwasaki T Nishikawa T Amagai M. Dominant autoimmune epitopes recognized by pemphigus antibodies map to the N-terminal adhesive region of desmogleins. J Immunol (2001) 167(9):5439–48. 10.4049/jimmunol.167.9.5439 11673563

[80] Futei Y Amagai M Sekiguchi M Nishifuji K Fujii Y Nishikawa T. Use of domain-swapped molecules for conformational epitope mapping of desmoglein 3 in pemphigus vulgaris. J Invest Dermatol (2000) 115(5):829–34. 10.1046/j.1523-1747.2000.00137.x 11069620

[81] Payne AS Ishii K Kacir S Lin C Li H Hanakawa Y Genetic and functional characterization of human pemphigus vulgaris monoclonal autoantibodies isolated by phage display. J Clin Invest (2005) 115(4):888–9. 10.1172/JCI24185 15841178 PMC1070425

[82] Tsunoda K Ota T Aoki M Yamada T Nagai T Nakagawa T Induction of pemphigus phenotype by a mouse monoclonal antibody against the amino-terminal adhesive interface of desmoglein 3. J Immunol (2003) 170(4):2170–8. 10.4049/jimmunol.170.4.2170 12574390

[83] Nguyen VT Ndoye A Grando SA Novel human alpha 9 acetylcholine receptor regulating keratinocyte adhesion is targeted by Pemphigus vulgaris autoimmunity. Am J Pathol (2000) 157(4):1377–91. 10.1016/j.bcp.2009.05.020 11021840 PMC1850172

[84] Nguyen VT Ndoye A Grando SA Pemphigus vulgaris antibody identifies pemphaxin. A novel keratinocyte annexinlike molecule binding acetylcholine. J Biol Chem (2000) 275(38):29466–76. 10.1074/jbc.M003174200 10899159

[85] Ahmed AR Carrozzo M Caux F Cirillo N Dmochowski M Alonso AE Monopathogenic vs multipathogenic explanations of pemphigus pathophysiology. Exp Dermatol (2016) 25(11):839–46. 10.1111/exd.13106 27305362

[86] Marchenko S Chernyavsky AI Arredondo J Gindi V Grando SA. Antimitochondrial autoantibodies in pemphigus vulgaris: a missing link in disease pathophysiology. J Biol Chem (2010) 285(6):3695–704. 10.1074/jbc.M109.081570 20007702 PMC2823510

[87] Calabresi V Mariotti F Pacifico V Didona B Zambruno G Di Zenzo G Reactivity against non disease-specific antigens in autoimmune bullous disease is rare and may represent an epiphenomenon. ESDR Annual Meeting; 2014 September 11-13; Copenhagen, Denmark. J Invest Dermatol (2014) 134:s14.

[88] Cho MJ Ellebrecht CT Hammers CM Mukherjee EM Sapparapu G Boudreaux CE Determinants of VH1-46 cross-reactivity to pemphigus vulgaris autoantigen desmoglein 3 and rotavirus antigen VP6. J Immunol (2016) 197(4):1065–73. 10.4049/jimmunol.1600567 27402694 PMC4976025

[89] Stanley JR Koulu L Thivolet C. Distinction between epidermal antigens binding pemphigus vulgaris and pemphigus foliaceus autoantibodies. J Clin Invest (1984) 74:313–20. 10.1172/JCI111426 6378972 PMC370481

[90] Diaz LA Sampaio SAP Rivitti EA Martins CR Cunha PR Lombardi C Endemic pemphigus foliaceus (fogo selvagem): II. Current and historic epidemiologic studies. J Invest Dermatol (1989) 92(1):4–12. 10.1111/1523-1747.ep13070394 2642512

[91] Abrèu-Velez AM Hashimoto T Bollag WB Tobón Arroyave S Abrèu-Velez CE Londoño ML A unique form of endemic pemphigus in northern Colombia. J Am Acad Dermatol (2003) 49(4):599–608. 10.1067/S0190-9622(03)00851-X 14512903

[92] Di Zenzo G Zambruno G Borradori L. Endemic pemphigus foliaceus: towards understanding autoimmune mechanisms of disease development. J Invest Dermatol (2012) 132:2499–502. 10.1038/jid.2012.369 23069908

[93] Ishii K Lin C Siegel DL Stanley JR. Isolation of pathogenic monoclonal anti-desmoglein 1 human antibodies by phage display of pemphigus foliaceus autoantibodies. J Invest Dermatol (2008) 128(4):939–48. 10.1038/sj.jid.5701132 18007588 PMC2597791

[94] Yokouchi M Saleh MA Kuroda K Hachiya T Stanley JR Amagai M Pathogenic epitopes of autoantibodies in pemphigus reside in the amino-terminal adhesive region of desmogleins which are unmasked by proteolytic processing of prosequence. J Invest Dermatol (2009) 129(9):2156–66. 10.1038/jid.2009.61 19340014 PMC2813511

[95] Sharma PM Choi EJ Kuroda K Hachiya T Ishii K Payne AS Pathogenic anti-desmoglein MAbs show variable ELISA activity because of preferential binding of mature versus proprotein isoforms of desmoglein 3. J Invest Dermatol (2009) 129(9):2309–12. 10.1038/jid.2009.41 19282843 PMC2841796

[96] Li N Aoki V Hans-Filho G Rivitti EA Diaz LA. The role of intramolecular epitope spreading in the pathogenesis of endemic pemphigus foliaceus (fogoselvagem). J Exp Med (2003) 197(11):1501–10. 10.1084/jem.20022031 12771179 PMC2193910

[97] Warren SJ Arteaga LA Rivitti EA Aoki V Hans-Filho G Qaqish BF The role of subclass switching in the pathogenesis of endemic pemphigus foliaceus. J Invest Dermatol (2003) 120:104–8. 10.1046/j.1523-1747.2003.12017.x 12535205

[98] Aoki V Rivitti EA Diaz LA. Update on fogo selvagem, an endemic form of pemphigus foliaceus. J Dermatol (2015) 42(1):18–26. 10.1111/1346-8138.12675 25558948 PMC4496802

[99] Aoki V Millikan RC Rivitti EA Hans-Filho G Eaton DP Warren SJ Environmental risk factors in endemic pemphigus foliaceus (fogo selvagem). J Investig Dermatol Symp Proc (2004) 9(1):34–40. 10.1111/j.1087-0024.2004.00833.x 14870983

[100] Qian Y Jeong JS Maldonado M Valenzuela JG Gomes R Teixeira C Cutting edge: brazilian pemphigus foliaceus anti-desmoglein 1 autoantibodies cross-react with sand fly salivary LJM11. Antigen J Immunol (2012) 189(4):1535–9. 10.4049/jimmunol.1200842 22798673 PMC3411885

[101] Qian Y Jeong JS Abdeladhim M Valenzuela JG Aoki V Hans-Filhio G IgE anti-LJM11 sand fly salivary antigen may herald the onset of Fogo selvagem in endemic Brazilian regions. J Invest Dermatol (2015) 135(3):913–5. 10.1038/jid.2014.430 25285921 PMC4323842

[102] Qian Y Jeong JS Ye J Dang B Abdeladhim M Aoki V Overlapping IgG4 responses to self- and environmental antigens in endemic pemphigus foliaceus. J Immunol (2016) 196(5):2041–50. 10.4049/jimmunol.1502233 26826247 PMC4761459

[103] Evangelista F Roth AJ Prisayanh P Temple BR Li N Qian Y Pathogenic IgG4 autoantibodies from endemic pemphigus foliaceus recognize a desmoglein-1 conformational epitope. J Autoimmun (2018). 10.1016/j.jaut.2017.12.017 29307589 PMC5902409

[104] Iwatsuki K Takigawa M Hashimoto T Nishikawa T Yamada M. Can pemphigus vulgaris become pemphigus foliaceus? J Am Acad Dermatol (1991) 25(5 Pt 1):797–800. 10.1016/S0190-9622(08)80971-1 1802901

[105] Kawana S Hashimoto T Nishikawa T Nishiyama S Shift in clinical features, histologic findings and antigen profiles from pemphigus vulgaris to pemphigus foliaceus – two case studies. Dermatology (1994) 189(Suppl 1):57–9. 10.1159/000246931 8049565

[106] Chang SN Kim SC Lee IJ Seo SJ Hong CK Park WH Transition from pemphigus vulgaris to pemphigus foliaceus. Br J Dermatol (1997) 137(2):303–5. 9292088 10.1046/j.1365-2133.1997.d01-2107.x

[107] Mendiratta V Sarkar R Sharma RC Korann RV. Transition of pemphigus vulgaris to pemphigus foliaceus. Indian J Dermatol Venereol Leprol (2000) 66(2):85–6. 20877034

[108] Ishii K Amagai M Ohata Y Shimizu H Hashimoto T Ohya K Development of pemphigus vulgaris in a patient with pemphigus foliaceus: antidesmoglein antibody profile shift confirmed by enzyme-linked immunosorbent assay. J Am Acad Dermatol (2000) 42(5 Pt 2):859–61. 10.1016/S0190-9622(00)90253-6 10767686

[109] Komai A Amagai M Ishii K Nishikawa T Chorzelski T Matsuo I The clinical transition between pemphigus foliaceus and pemphigus vulgaris correlates well with the changes in autoantibody profile assessed by an enzyme-linked immunosorbent assay. Br J Dermatol (2001) 144(6):1177–82. 10.1046/j.1365-2133.2001.04227.x 11422038

[110] Kimoto M Ohyama M Hata Y Amagai M Nishikawa T. A case of pemphigus foliaceus which occurred after five years of remission from pemphigus vulgaris. Dermatology (2001) 203(2):174–6. 10.1159/000051737 11586021

[111] Tsuji Y Kawashima T Yokota K Tateish Y Tomita Y Matsumura T Clinical and serological transition from pemphigus vulgaris to pemphigus foliaceus demonstrated by desmoglein ELISA system. Arch Dermatol (2002) 138(1):95–6. 10.1001/archderm.138.1.95 11790172

[112] Harman KE Gratian MJ Shirlaw PJ Bhogal BS Challacombe SJ Black MM. The transition of pemphigus vulgaris into pemphigus foliaceus: a reflection of changing desmoglein 1 and 3 autoantibody levels in pemphigus vulgaris. Br J Dermatol (2002) 146(4):684–7. 10.1046/j.1365-2133.2002.04608.x 11966706

[113] Tóth GG Pas HH Jonkman MF Transition of pemphigus vulgaris into pemphigus foliaceus confirmed by antidesmoglein ELISA profile. Int J Dermatol (2002) 41(8):525–7. 10.1046/j.1365-4362.2002.15452.x 12207776

[114] Ng PP Thng ST. Three cases of transition from pemphigus vulgaris to pemphigus foliaceus confirmed by desmoglein ELISA. Dermatology (2005) 210(4):319–21. 10.1159/000084757 15942219

[115] Park SG Chang JY Cho YH Kim SC Lee MG. Transition from pemphigus foliaceus to pemphigus vulgaris: case report with literature review. Yonsei Med J (2006) 47(2):278–81. 10.3349/ymj.2006.47.2.278 16642562 PMC2687642

[116] Awazawa R Yamamoto Y Gushi M Taira K Yagi N Asato Y Case of pemphigus foliaceus that shifted into pemphigus vulgaris after adrenal tumor resection. J Dermatol (2007) 34(8):549–55. 10.1111/j.1346-8138.2007.00329.x 17683386

[117] Pigozzi B Peserico A Schiesari L Alaibac M Pemphigus foliaceus evolving into pemphigus vulgaris: a probable example of ‘intermolecular epitope spreading’ confirmed by enzyme-linked immunosorbent assay study. J Eur Acad Dermatol Venereol (2008) 22(2):242–4. 10.1111/j.1468-3083.2007.02298.x 18211424

[118] Lévy-Sitbon C Reguiaï Z Durlach A Goeldel AL Grange F Bernard P Transition from pemphigus vulgaris to pemphigus foliaceus: a case report. Ann Dermatol Venereol (2013) 140(12):788–92. 10.1016/j.annder.2013.07.013 24315225

[119] España A Koga H Suárez-Fernández R Ohata C Ishii N Irarrazaval I Antibodies to the amino-terminal domain of desmoglein 1 are retained during transition from pemphigus vulgaris to pemphigus foliaceus. Eur J Dermatol (2014) 24(2):174–9. 10.1684/ejd.2014.2277 24776707

[120] Ito T Moriuchi R Kikuchi K Shimizu S Rapid transition from pemphigus vulgaris to pemphigus foliaceus. J Eur Acad Dermatol Venereol (2016) 30(3):455–7. 10.1111/jdv.12832 25376758

[121] Sami N Ahmed AR. Dual diagnosis of pemphigus and pemphigoid. Retrospective review of thirty cases in the literature. Dermatology (2001) 202(4):293–301. 10.1159/000051661 11455140

[122] Korman NJ Stanley JR Woodley DT. Coexistence of pemphigus foliaceus and bullous pemphigoid. Demonstration of autoantibodies that bind to both the pemphigus foliaceus antigen complex and the bullous pemphigoid antigen. Arch Dermatol (1991) 127(3):387–90. 10.1001/archderm.127.3.387 1998370

[123] Peterson JD Chang AJ Chan LS Clinical evidence of an intermolecular epitope spreading in a patient with pemphigus foliaceus converting into bullous pemphigoid. Arch Dermatol (2007) 143(2):272–4. 10.1001/archderm.143.2.272 17310017

[124] Recke A Rose C Schmidt E Bröcker EB Zillikens D Sitaru C. Transition from pemphigus foliaceus to bullous pemphigoid: intermolecular B-cell epitope spreading without IgG subclass shifting. J Am Acad Dermatol (2009) 61(2):333–6. 10.1016/j.jaad.2008.10.061 19615544

[125] Didona D Didona B Richetta AG Cantisani C Moliterni E Calvieri S Paraneoplastic pemphigus: a trait d’union between dermatology and oncology. Adv Mod Oncol Res (2015) 1(2):97–103. 10.18282/amor.v1.i2.42

[126] Paolino G Didona D Magliulo G Iannella G Didona B Mercuri SR Paraneoplastic pemphigus: insight into the autoimmune pathogenesis, clinical features and therapy. Int J Mol Sci (2017) 18(12):E2532. 10.3390/ijms18122532 29186863 PMC5751135

[127] Schepens I Jaunin F Begre N Läderach U Marcus K Hashimoto T The protease inhibitor alpha-2-macroglobulin-like-1 is the p170 antigen recognized by paraneoplastic pemphigus autoantibodies in human. PLoS One (2010) 5(8):e12250. 10.1371/journal.pone.0012250 20805888 PMC2923615

[128] Mimouni D Foedinger D Kouba DJ Orlow SJ Rappersberger K Sciubba JJ Mucosal dominant pemphigus vulgaris with anti-desmoplakin autoantibodies. J Am Acad Dermatol (2004) 51(1):62–7. 10.1016/j.jaad.2003.11.051 15243525

[129] Cozzani E Dal Bello MG Mastrogiacomo A Drosera M Parodi A. Antidesmoplakin antibodies in pemphigus vulgaris. Br J Dermatol (2006) 154(4):624–8. 10.1111/j.1365-2133.2005.06987.x 16536803

[130] Ortolan DG Souza DP Aoki V Santi CG Gabbi TV Ichimura LM Analysis of the reactivity of indirect immunofluorescence in patients with pemphigus foliaceus and pemphigus vulgaris using rat bladder epithelium as a substrate. Clinics (Sao Paulo) (2011) 66(12):2019–23. 10.1590/S1807-59322011001200004 22189724 PMC3226594

[131] Watanabe T Kato M Yoshida Y Fukuda S Hashimoto T Yamamoto O Mucocutaneous-type pemphigus vulgaris with anti-desmoplakin autoantibodies. Eur J Dermatol (2011) 21(2):299–300. 10.1684/ejd.2011.1307 21524991

[132] Bowen GM Peters NT Fivenson DP Su LD Nousari HC Anhalt GJ Lichenoid dermatitis in paraneoplastic pemphigus: a pathogenic trigger of epitope spreading? Arch Dermatol (2000) 136(5):652–6. 10.1001/archderm.136.5.652 10815859

[133] Okahashi K Oiso N Ishii N Miyake M Uchida S Matsuda H Paraneoplastic pemphigus associated with Castleman disease: progression from mucous to mucocutaneous lesions with epitope-spreading phenomena. Br J Dermatol (2017) 176(5):1406–9. 10.1111/bjd.15389 28213962

[134] Saleh MA Ishii K Yamagami J Shirakata Y Hashimoto K Amagai M. Pathogenic anti-desmoglein 3 mAbs cloned from a paraneoplastic pemphigus patient by phage display. J Invest Dermatol (2012) 132(4):1141–8. 10.1038/jid.2011.449 22277944

[135] Futei Y Amagai M Hashimoto T Nishikawa T. Conformational epitope mapping and IgG subclass distribution of desmoglein 3 in paraneoplastic pemphigus. J Am Acad Dermatol (2003) 49(6):1023–8. 10.1016/s0190 14639380

[136] Robinson N Hashimoto T Amagai M Chan LS. The new pemphigus variants. J Am Acad Dermatol (1999) 40(5 Pt 1):649–71. quiz 672-3, 10.1016/S0190-9622(99)70145-3 10321591

[137] Woodley DT Briggaman RA O’Keefe EJ Inman AO Queen LL Gammon WR. Identification of the skin basement-membrane autoantigen in epidermolysis bullosa acquisita. N Engl J Med (1984) 310(16):1007–13. 10.1056/NEJM198404193101602 6369131

[138] Gammon WR. Epidermolysis bullosa acquisita: a disease of autoimmunity to type VII collagen. J Autoimmun (1991) 4(1):59–71. 10.1016/0896-8411(91)90007-Y 2031664

[139] Calabresi V Sinistro A Cozzani E Cerasaro C Lolicato F Muscianese M Sensitivity of different assays for the serological diagnosis of epidermolysis bullosa acquisita: analysis of a cohort of 24 Italian patients. J Eur Acad Dermatol Venereol (2014) 28(4):483–90. 10.1111/jdv.12129 24321031

[140] Ludwig RJ Recke A Bieber K Müller S Marques Ade C Banczyk D Generation of antibodies of distinct subclasses and specificity is linked to H2s in an active mouse model of epidermolysis bullosa acquisita. J Invest Dermatol (2011) 131(1):167–76. 10.1038/jid.2010.248 20720563

[141] Gammon WR Briggaman RA Epidermolysis bullosa acquisita and bullous systemic lupus erythematosus. Dermat Clin (1993) 11(3):535–47. 8365038

[142] Lapiere JC Woodley DT Parente MG Iwasaki T Wynn KC Christiano AM Epitope mapping of type VII collagen. Identification of discrete peptide sequences recognized by sera from patients with acquired epidermolysis bullosa. J Clin Invest (1993) 92(4):1831–9. 10.1172/JCI116774 7691888 PMC288347

[143] Chan LS Lapiere JC Chen M Traczyk T Mancini AJ Paller AS Bullous systemic lupus erythematosus with autoantibodies recognizing multiple skin basement membrane components, bullous pemphigoid antigen 1, laminin-5, laminin-6, and type VII collagen. Arch Dermatol (1999) 135(5):569–73. 10.1001/archderm.135.5.569 10328198

[144] Chen M Marinkovich MP Jones JC O’Toole EA Li YY Woodley DT. NC1 domain of type VII collagen binds to the beta3 chain of laminin 5 via a unique subdomain within the fibronectin-like repeats. J Invest Dermatol (1999) 112(2):177–83. 10.1046/j.1523-1747.1999.00491.x 9989793

[145] Dotson AD Raimer SS Pursley TV Tschen J. Systemic lupus erythematosus occurring in a patient with epidermolysis bullosa acquisita. Arch Dermatol (1981) 117(7):422–6. 10.1001/archderm.1981.01650070050025 7259221

[146] Barton DD Fine JD Gammon WR Sams WM Jr. Bullous systemic lupus erythematosus: an unusual clinical course and detectable circulating autoantibodies to the epidermolysis bullosa acquisita antigen. J Am Acad Dermatol (1986) 15(2 Pt 2):369–73. 10.1016/S0190-9622(86)70181-3 3525621

[147] Kettler AH Bean SF Duffy JO Gammon WR. Systemic lupus erythematosus presenting as a bullous eruption in a child. Arch Dermatol (1988) 124(7):1083–7. 10.1001/archderm.124.7.1083 3291780

[148] Boh E Roberts LJ Lieu TS Gammon WR Sontheimer RD. Epidermolysis bullosa acquisita preceding the development of systemic lupus erythematosus. J Am Acad Dermatol (1990) 22(4):587–93. 10.1016/0190-9622(90)70077-U 2319019

[149] McHenry PM Dagg JH Tidman MJ Lever RS. Epidermolysis bullosa acquisita occurring in association with systemic lupus erythematosus. Clin Exp Dermatol (1993) 18(4):378–80. 10.1111/j.1365-2230.1993.tb02224.x 8403483

[150] Yoon J Moon TK Lee KH Kim SC. Fatal vascular involvement in systemic lupus erythematosus following epidermolysis bullosa acquisita. Acta Derm Venereol (1995) 75(2):143–6. 7604645 10.2340/0001555575143146

[151] Chen M O’Toole EA Sanghavi J Mahmud N Weir D Kelleher D Type VII collagen exists in human intestine and serves as an antigenic target in patients with inflammatory bowel disease. J Eur Acad Dermatol Venereol (1997) 108:542.

[152] Licarete E Ganz S Recknagel M Di Zenzo G Hashimoto T Hertl M Prevalence of collagen VII-specific autoantibodies in patients with autoimmune and inflammatory diseases. BMC Immunol (2012) 13:16. 10.1186/1471-2172-13-16 22471736 PMC3368718

[153] Lohi J Leivo I Tani T Kiviluoto T Kivilaakso E Burgeson RE Laminins, tenascin and type VII collagen in colorectal mucosa. Histochem J (1996) 28(6):431–40. 10.1007/BF02331434 8863048

[154] Chan LS Vanderlugt CJ Hashimoto T Nishikawa T Zones JJ Black MM Epitope spreading: lessons from autoimmune skin diseases. J Invest Dermatol (1998) 110(2):103–9. 10.1046/j.1523-1747.1998.00107.x 9457902

[155] Kirtschig G Chow ET Venning VA Wojnarowska FT. Acquired subepidermal bullous diseases associated with psoriasis: a clinical, immunopathological and immunogenetic study. Br J Dermatol (1996) 135(5):738–45. 10.1111/j.1365-2133.1996.tb03883.x 8977674

[156] Saeki H Hayashi N Komine M Soma Y Shimada S Watanabe K A case of generalized pustular psoriasis followed by bullous disease: an atypical case of bullous pemphigoid or a novel bullous disease? Br J Dermatol (1996) 134(1):152–5. 10.1046/j.1365-2133.1996.d01-758.x 8745904

[157] Endo Y Tamura A Ishikawa O Miyachi Y Hashimoto T. Psoriasis vulgaris coexistent with epidermolysis bullosa acquisita. Br J Dermatol (1997) 137(5):783–6. 10.1111/j.1365-2133.1997.tb01119.x 9415242

[158] Hoshina D Sawamura D Nomura T Tanimura S Abe M Onozuka T Epidermolysis bullosa acquisita associated with psoriasis vulgaris. Clin Exp Dermatol (2007) 32(5):516–8. 10.1111/j.1365-2230.2007.02430.x 17459071

[159] Kabashima R Hino R Bito T Kabashima K Nakamura M Bungo O Epidermolysis bullosa acquisita associated with psoriasis. Acta Derm Venereol (2010) 90(3):314–6. 10.2340/00015555-0832 20526561

[160] Min L Kensuke M Takashi H Naoyuki H Epidermolysis bullosa acquisita in a patient with psoriasis vulgaris. Eur J Dermatol (2015) 25(5):499–500. 10.1684/ejd.2015.2623 26243636

[161] Moon SY Eun DH Jung HJ Kim JY Park TI Lee WJ Coexistence of psoriasis and epidermolysis bullosa acquisita: evaluation of the integrity of the basement membrane. J Cutan Pathol (2017) 44(6):602–3. 10.1111/cup.12940 28425108

[162] Kawachi Y Ikegami M Hashimoto T Matsumura K Tanaka T Otsuka F. Autoantibodies to bullous pemphigoid and epidermolysis bullosa acquisita antigens in an infant. Br J Dermatol (1996) 135(3):443–7. 10.1111/j.1365-2133.1996.tb01511.x 8949441

[163] Jonkman MF Schuur J Dijk F Heeres K de Jong MC van der Meer JB Inflammatory variant of epidermolysis bullosa acquisita with IgG autoantibodies against type VII collagen and laminin alpha3. Arch Dermatol (2000) 136(2):227–31. 10.1001/archderm.136.2.227 10677099

[164] Furukawa H Miura T Takahashi M Nakamura K Kaneko F Ishii F A case of anti-p200 pemphigoid with autoantibodies against both a novel 200-kD dermal antigen and the 290-kD epidermolysis bullosa acquisita antigen. Dermatology (2004) 209(2):145–8. 10.1159/000079601 15316171

[165] Buijsrogge JJ de Jong MC Meijer HJ Dijk F Jonkman MF Pas HH. Inflammatory epidermolysis bullosa acquisita with coexistent IgA antibodies to plectin. Clin Exp Dermatol (2005) 30(5):531–4. 10.1111/j.1365-2230.2005.01854.x 16045687

[166] Osawa M Demitsu T Toda S Yokokura H Umemoto N Yamada T A case of mixed bullous disease of epidermolysis bullosa acquisita and linear IgA bullous dermatosis. Dermatology (2005) 211(2):146–8. 10.1159/000086445 16088162

[167] Yang B Wang C Wu M Du D Yan X Zhou G A case of pemphigoid gestationis with concurrent IgG antibodies to BP180, BP230 and type VII collagen. Australas J Dermatol (2014) 55(1):e15–8. 10.1111/j.1440-0960.2012.00960.x 23082779

[168] Marathe K Lu J Morel KD. Bullous diseases: kids are not just little people. Clin Dermatol (2015) 33(6):644–56. 10.1016/j.clindermatol.2015.09.007 26686016

[169] Allen J Wojnarowska F. Linear IgA disease: the IgA and IgG response to dermal antigens demonstrates a chiefly IgA response to LAD285 and a dermal 180-kDa protein. Br J Dermatol (2003) 149(5):1055–8. 10.1111/j.1365-2133.2003.05647.x 14632815

[170] Allen J Wojnarowska F. Linear IgA disease: the IgA and IgG response to the epidermal antigens demonstrates that intermolecular epitope spreading is associated with IgA rather than IgG antibodies, and is more common in adults. Br J Dermatol (2003) 149(5):977–85. 10.1111/j.1365-2133.2003.05648.x 14632802

[171] Zillikens D Herzele K Georgi M Schmidt E Chimanovitch I Schumann H Autoantibodies in a subgroup of patients with linear IgA disease react with the NC16A domain of BP180. J Invest Dermatol (1999) 113(6):947–53. 10.1046/j.1523-1747.1999.00808.x 10594735

[172] Marinkovich P Taylor T Keene D Burgeson RE Zone JJ LAD-1, the linear IgA bullous dermatosis autoantigen, is a novel 120kDa anchoring filament protein synthesized by epidermal cells. J Invest Dermatol (1996) 106(4):734–8. 10.1111/1523-1747.ep12345782 8618013

[173] Zone JJ Taylor TB Meyer LJ Petersen MJ. The 97 kDa linear IgA bullous disease antigen is identical to a portion of the extracellular domain of the 180 kDa bullous pemphigoid antigen, BPAg2. J Invest Dermatol (1998) 110(3):207–10. 10.1046/j.1523-1747.1998.00129.x 9506436

[174] Roh JY Yee C Lazarova Z Hall RP Yancey KB. The 120-kDa soluble ectodomain of type XVII collagen is recognized by autoantibodies in patients with pemphigoid and linear IgA dermatosis. Br J Dermatol (2000) 143(1):104–11. 10.1046/j.1365-2133.2000.03598.x 10886143

[175] Christophoridis S Budinger L Borradori L Hunziker T Merk HF Hertl M. IgG, IgA and IgE autoantibodies against the ectodomain of BP180 in patients with bullous and cicatricial pemphigoid, and linear IgA bullous dermatosis. Br J Dermatol (2000) 143(2):349–55. 10.1046/j.1365-2133.2000.03661.x 10951144

[176] Sakaguchi M Bito T Oda Y Kikusawa A Nishigori C Munetsugu T Three cases of linear IgA/IgG bullous dermatosis showing IgA and IgG reactivity with multiple antigens, particularly laminin-332. JAMA Dermatol (2013) 149(11):1308–13. 10.1001/jamadermatol.2013.5691 24005769

[177] Kanitakis J Mauduit G Cozzani E Badinand P Faure M Claudy A. Linear IgA bullous dermatosis of childhood with autoantibodies to a 230 kDa epidermal antigen. Pediatr Dermatol (1994) 11(2):139–44. 10.1111/j.1525-1470.1994.tb00568.x 8041654

[178] Zambruno G Manca V Kanitakis J Cozzani E Nicolas JF Giannetti A. Linear IgA bullous dermatosis with autoantibodies to a 290 kd antigen of anchoring fibrils. J Am Acad Dermatol (1994) 31(5 Pt 2):884–8. 10.1016/S0190-9622(94)70252-7 7962741

[179] Bérard F Kanitakis J Di Maio M Ghohestani R Hermier C David L Linear IgA bullous dermatosis in children with autoantibodies against 180 kDa pemphigoid antigen. Arch Pediatr (1996) 3(4):345–7. 8762956 10.1016/0929-693x(96)84689-6

[180] Hashimoto T Ishiko A Shimizu H Tanaka T Dodd HJ Bhogal BS A case of linear IgA bullous dermatosis with IgA anti-type VII collagen autoantibodies. Br J Dermatol (1996) 134(2):336–9. 10.1111/j.1365-2133.1996.tb07624.x 8746352

[181] Kawahara Y Hashimoto T Watanabe K Kurihara S Matsuo I Nishikawa T. Two cases of atypical bullous disease showing linear IgG and IgA deposition in the basement membrane zone. J Dermatol (1996) 23(4):254–8. 10.1111/j.1346-8138.1996.tb04008.x 8935340

[182] Ghohestani RF Nicolas JF Kanitakis J Claudy A. Linear IgA bullous dermatosis with IgA antibodies exclusively directed against the 180- or 230-kDa epidermal antigens. J Invest Dermatol (1997) 108(6):854–8. 10.1111/1523-1747.ep12292581 9182810

[183] Honoki K Muramatsu T Tsubakimoto A Shirai T. Linear IgA bullous dermatosis with circulating IgG autoantibodies to the 230 kD epidermal antigen. J Dermatol (1998) 25(8):503–9. 10.1111/j.1346-8138.1998.tb02444.x 9769594

[184] Wakelin SH Allen J Zhou S Wojnarowska F. Drug-induced linear IgA disease with antibodies to collagen VII. Br J Dermatol (1998) 138(2):310–4. 10.1046/j.1365-2133.1998.02081.x 9602881

[185] Nie Z Nagata Y Joubeh S Hirako Y Owaribe K Kitajima Y IgA antibodies of linear IgA bullous dermatosis recognize the 15th collagenous domain of BP180. J Invest Dermatol (2000) 115(6):1164–6. 10.1046/j.1523-1747.2000.0202a-7.x 11121162

[186] Lin MS Fu CL Olague-Marchan M Hacker MK Zillikens D Giudice GJ Autoimmune responses in patients with linear IgA bullous dermatosis: both autoantibodies and T lymphocytes recognize the NC16A domain of the BP180 molecule. Clin Immunol (2002) 102(3):310–9. 10.1006/clim.2001.5177 11890718

[187] Metz BJ Ruggeri SY Hsu S Reed JA Ghohestani AS Uitto J Linear IgA dermatosis with IgA and IgG autoantibodies to the 180 kDa bullous pemphigoid antigen (BP180): evidence for a distinct subtype. Int J Dermatol (2004) 43(6):443–6. 10.1111/j.1365-4632.2004.02016.x 15186228

[188] Shimizu S Natsuga K Shinkuma S Yasui C Tsuchiya K Shimizu H. Localized linear IgA/IgG bullous dermatosis. Acta Derm Venereol (2010) 90(6):621–4. 10.2340/00015555-0985 21057747

[189] Passos L Rabelo RF Matsuo C Santos M Talhari S Talhari C. Linear IgA/IgG bullous dermatosis: successful treatment with dapsone and mycophenolate mofetil. An Bras Dermatol (2011) 86(4):747–50. 10.1590/S0365-05962011000400018 21987142

[190] Yanagihara S Mizuno N Naruse A Tateishi C Tsuruta D Ishii M. Linear immunoglobulin A/immunoglobulin G bullous dermatosis associated with Vogt-Koyanagi-Harada disease. J Dermatol (2011) 38(8):798–791. 10.1111/j.1346-8138.2011.01221.x 21545501

[191] Kern JS Gehring W Kreisel W Hertl M Technau-Hafsi K Bruckner-Tuderman L Overlap of IgA pemphigus and linear IgA dermatosis in a patient with ulcerative colitis: a mere coincidence? Acta Derm Venereol (2014) 94(2):228–30. 10.2340/00015555-1658 23824334

[192] Tashima S Konishi K Koga H Hashimoto T A case of vancomycin-induced linear IgA bullous dermatosis with circulating IgA antibodies to the NC16a domain of BP180. Int J Dermatol (2014) 53(3):e207–9. 10.1111/ijd.12047 23829415

[193] Zenke Y Nakano T Eto H Koga H Hashimoto T. A case of vancomycin-associated linear IgA bullous dermatosis and IgA antibodies to the α3 subunit of laminin-332. Br J Dermatol (2014) 170(4):965–9. 10.1111/bjd.12720 24641255

[194] Izaki S Mitsuya J Okada T Koga H Hashimoto T Terui T A case of linear IgA/IgG bullous dermatosis with anti-laminin-332 autoantibodies. Acta Derm Venereol (2015) 95(3):359–60. 10.2340/00015555-1923 24978847

[195] Li X Tsuchisaka A Qian H Teye K Ishii N Sogame R Linear IgA/IgG bullous dermatosis reacts with multiple laminins and integrins. Eur J Dermatol (2015) 25(5):418–23. 10.1684/ejd.2015.2555 26069157

[196] Fernandes KA Galvis KH Gomes AC Nogueira OM Felix PA Vargas TJ. Linear IgA and IgG bullous dermatosis. An Bras Dermatol (2016) 91(5 Suppl 1):32–4. 10.1590/abd1806-4841.20164630 28300887 PMC5324986

[197] Izaki S Ito K Ishii N Hashimoto T Fujita H Terui T Infantile linear IgA/IgG bullous dermatosis. Eur J Dermatol (2016) 26(1):96–8. 10.1684/ejd.2015.2667 26553506

[198] Koga H Ishii N Hashimoto T Nakama T. Case of shift from linear immunoglobulin A bullous dermatosis to pemphigus herpetiformis for a short period of time. J Dermatol (2017) 44(2):189–93. 10.1111/1346-8138.13677 28497856

[199] Matsuura K Ujiie H Hayashi M Muramatsu K Yoshizawa J Ito T Linear IgA bullous dermatosis in a pregnant woman with autoantibodies to the non-collagenous 16A domain of type XVII collagen. Acta Derm Venereol (2017) 97(3):404–5. 10.2340/00015555-2557 27786348

[200] Cooke N Jenkinson H Wojnarowska F McKenna K Alderdice J. Coexistence of psoriasis and linear IgA disease in a patient with recent herpes zoster infection. Clin Exp Dermatol (2005) 30(6):643–5. 10.1111/j.1365-2230.2005.01872.x 16197377

[201] Takagi Y Sawada S Yamauchi M Amagai M Niimura M. Coexistence of psoriasis and linear IgA bullous dermatosis. Br J Dermatol (2000) 142(3):513–6. 10.1046/j.1365-2133.2000.03367.x 10735961

[202] Barrows-Wade L Jordon RE Arnett FC Jr Linear IgA bullous dermatosis associated with dermatomyositis. Arch Dermatol (1992) 128(3):413–4. 10.1001/archderm.128.3.413 1550384

[203] Hayakawa K Shiohara T Yagita A Nagashima M. Linear IgA bullous dermatosis associated with rheumatoid arthritis. J Am Acad Dermatol (1992) 26(1):110–3. 10.1016/0190-9622(92)70017-A 1732316

[204] Alba D Alvarez-Doforno R Casado M Borbujo J Linear bullous IGA dermatosis and systemic lupus erythematosus. Med Clin (Barc) (1995) 105(2):77–8. 7603102

[205] Tobón GJ Toro CE Bravo JC Cañas CA. Linear IgA bullous dermatosis associated with systemic lupus erythematosus: a case report. Clin Rheumatol (2008) 27(3):391–3. 10.1007/s10067-007-0752-5 17932615

[206] Mavragani CP Asvesti K Moutsopoulos HM Linear IgA dermatosis in a patient with primary Sjogren’s syndrome. Rheumatology (Oxford) (2013) 52(2):403–4. 10.1093/rheumatology/kes148 22763990

[207] Paige DG Leonard JN Wojnarowska F Fry L. Linear IgA disease and ulcerative colitis. Br J Dermatol (1997) 136(5):779–82. 10.1046/j.1365-2133.1997.6751622.x 9205518

[208] Chan LS Regezi JA Cooper KD. Oral manifestations of linear IgA disease. J Am Acad Dermatol (1990) 22(2 Pt 2):362–5. 10.1016/0190-9622(90)70049-N 2406301

[209] De Simone C Guerriero C Pellicano R. Linear IgA disease and ulcerative colitis. Eur J Dermatol (1998) 8(1):48–50. 9649655

[210] Chi HI Arai M. Linear IgA bullous dermatosis associated with ulcerative colitis. J Dermatol (1999) 26(3):150–3. 10.1111/j.1346-8138.1999.tb03445.x 10209920

[211] Keller AS Bouldin MB Drage LA Hauser SC Davis MD Linear IgA bullous dermatosis: an association with ulcerative colitis versus renal cell carcinoma. Dig Dis Sci (2003) 48(4):783–9. 10.1023/A:1022805329847 12741472

[212] Eckert RL Sturniolo MT Broome AM Ruse M Rorke EA. Transglutaminase function in epidermis. J Invest Dermatol (2005) 124(3):481–92. 10.1111/j.0022-202X.2005.23627.x 15737187

[213] Kárpáti S Sárdy M Németh K Mayer B Smyth N Paulsson M Transglutaminases in autoimmune and inherited skin diseases: the phenomena of epitope spreading and functional compensation. Exp Dermatol (2017). 10.1111/exd.13449 28940785

[214] Mendes FB Hissa-Elian A Abreu MA Gonçalves VS. Review: dermatitis herpetiformis. An Bras Dermatol (2013) 88(4):594–5. 10.1590/abd1806-4841.20131775 24068131 PMC3760935

[215] Van der Meer JB Granular deposits of immunoglobulins in the skin of patients with dermatitis herpetiformis, an immunofluorescence study. Br J Dermatol (1969) 81(7):493–493. 10.1111/j.1365-2133.1969.tb16024.x 4183364

[216] Honeyman JF Honeyman A Lobitz WC Storrs FJ The enigma of bullous pemphigoid and dermatitis herpetiformis. Arch Dermatol (1972) 106(1):22–5. 10.1001/archderm.1972.01620100010002 4556949

[217] Jablonska S Chorzelski TP Beutner E Maciejowska E Rzsa G. Dermatitis herpetiformis and bullous pemphigoid. Intermediate and mixed forms. Arch Dermatol (1976) 112:45–8. 10.1001/archderm.112.1.45 1108803

[218] Bean SF Michel B Furey N Thorne EG Meltzer L. Vesicular pemphigoid. Arch Dermatol (1976) 112(10):1402–4. 10.1001/archderm.112.10.1402 786176

[219] Honeyman JF Honeyman AR De la Parra MA Pinto A Eguiguren GJ. Polymorphic pemphigoid. Arch Dermatol (1979) 115(4):423–7. 10.1001/archderm.115.4.423 373638

[220] Jolliffe DS Sarkany I Mixed bullous disease. Clin Exp Dermatol (1983) 8(1):113–6. 10.1111/j.1365-2230.1983.tb01752.x 6340866

[221] de Jong MC van der Meer JB de Nijs JA van der Putte SC. Concomitant immunohistochemical characteristics of pemphigoid and dermatitis herpetiformis in a patient with atypical bullous dermatosis. Acta Derm Venereol (1983) 63(6):476–82. 6198835

[222] Jawitz J Kumar V Nigra TP Beutner EH Vesicular pemphigoid versus dermatitis herpetiformis. J Am Acad Dermatol (1984) 10(5 Pt 2):892–6. 10.1016/S0190-9622(84)80441-7 6373860

[223] Sander HM Utz MMP Peters MS. Bullous pemphigoid and dermatitis herpetiformis: mixed bullous disease or coexistence of two separate entities? J Cutan Pathol (1989) 16(6):370–4. 10.1111/j.1600-0560.1989.tb00588.x 2693505

[224] Setterfield J Bhogal B Black MM McGibbon DH. Dermatitis herpetiformis and bullous pemphigoid: a developing association confirmed by immunoelectronmicroscopy. Br J Dermatol (1997) 136(2):253–6. 10.1046/j.1365-2133.1997.d01-1181.x 9068744

[225] Ameen M Bhogal BS Black MM Dermatitis herpetiformis evolving into bullous pemphigoid: a probable example of epitope spreading. Clin Exp Dermatol (2000) 25(5):398–400. 10.1046/j.1365-2230.2000.00673.x 11012594

[226] Murphy LA Bhogal BS Banerjee P Black MM Dermatitis herpetiformis converting into bullous pemphigoid: a study of three cases. Br J Dermatol (2003) 149(Suppl 64):17.12890190

[227] Schulze F van Beek N Terheyden P Zillikens D Schmidt E. Concomitant bullous pemphigoid and dermatitis herpetiformis. Dermatology (2013) 226(3):217–21. 10.1159/000349982 23775006

[228] Vaira F Della Valle V Fanoni D Pontini P Muratori S Bullous pemphigoid and dermatitis herpetiformis association: a genetic predisposition. J Dermatol (2013) 40(11):940–1. 10.1111/1346-8138.12262 24128323

[229] Chan LS. Ocular and oral mucous membrane pemphigoid (cicatricial pemphigoid). Clin Dermatol (2012) 30(1):34–7. 10.1016/j.clindermatol.2011.03.007 22137224

[230] Bruch-Gerharz D Hertl M Ruzicka T. Mucous membrane pemphigoid: clinical aspects, immunopathological features and therapy. Eur J Dermatol (2007) 17(3):191–200. 10.1684/ejd.2007.0148 17478379

[231] Balding SD Prost C Diaz LA Bernard P Bedane C Aberdam D Cicatricial pemphigoid autoantibodies react with multiple sites on the BP180 extracellular domain. J Invest Dermatol (1996) 106(1):141–6. 10.1111/1523-1747.ep12329728 8592065

[232] Calabresi V Carrozzo M Cozzani E Arduino P Bertolusso G Tirone F Oral pemphigoid autoantibodies preferentially target BP180 ectodomain. Clin Immunol (2007) 122:207–13. 10.1016/j.clim.2006.10.007 17141573

[233] Cozzani E Di Zenzo G Calabresi V Carrozzo M Burlando M Longanesi L Autoantibody profile of a cohort of 78 Italian patients with mucous membrane pemphigoid: correlation between reactivity profile and clinical involvement. Acta Derm Venereol (2016) 96:768–73. 10.2340/00015555-2311 26631393

[234] Murrell DF Marinovic B Caux F Prost C Ahmed R Wozniak K Definitions and outcome measures for mucous membrane pemphigoid: recommendations of an international panel of experts. J Am Acad Dermatol (2015) 72(1):168–74. 10.1016/j.jaad.2014.08.024 25443626

[235] Bernard P Antonicelli F Bedane C Joly P Le Roux-Villet C Duvert-Lehembre S Prevalence and clinical significance of anti-laminin 332 autoantibodies detected by a novel enzyme-linked immunosorbent assay in mucous membrane pemphigoid. JAMA Dermatol (2013) 149(5):533–40. 10.1001/jamadermatol.2013.1434 23426192

[236] Setterfield J Shirlaw PJ Kerr-Muir M Neill S Bhogal BS Morgan P Mucous membrane pemphigoid: a dual circulating antibody response with IgG and IgA signifies a more severe and persistent disease. Br J Dermatol (1998) 138(4):602–10. 10.1046/j.1365-2133.1998.02168.x 9640363

[237] Egan CA Hanif N Taylor TB Meyer LJ Petersen MJ Zone JJ. Characterization of the antibody response in oesophageal cicatricial pemphigoid. Br J Dermatol (1999) 140(5):859–64. 10.1046/j.1365-2133.1999.03159.x 10354023

[238] Yasukochi A Teye K Ishii N Hashimoto T. Clinical and immunological studies of 332 Japanese patients tentatively diagnosed as anti-BP180-type mucous membrane pemphigoid: a novel BP180 C-terminal domain enzyme-linked immunosorbent assay. Acta Derm Venereol (2016) 96(6):762–7. 10.2340/00015555-2407 26984589

[239] Lazarova Z Hsu R Yee C Yancey KB Human anti-laminin 5 autoantibodies induce subepidermal blisters in an experimental human skin graft model. J Invest Dermatol (2000) 114(1):178–84. 10.1046/j.1523-1747.2000.00829.x 10620135

[240] Oyama N Setterfield JF Powell AM Sakuma-Oyama Y Albert S Bhogal BS Bullous pemphigoid antigen II (BP180) and its soluble extracellular domains are major autoantigens in mucous membrane pemphigoid: the pathogenic relevance to HLA class II alleles and disease severity. Br J Dermatol (2006) 154(1):90–8. 10.1111/j.1365-2133.2005.06998.x 16403100

[241] Inoue T Yagami A Iwata Y Ishii N Hashimoto T Matsunaga K Mucous membrane pemphigoid reactive only with BP230. J Dermatol (2016) 43(10):1228–9. 10.1111/1346-8138.13361 27709732

[242] Hayashi I Shinkuma S Shimizu S Natsuga K Ujiie H Yasui C Mucous membrane pemphigoid with generalized blisters: IgA and IgG autoantibodies target both laminin-332 and type XVII collagen. Br J Dermatol (2012) 166(5):1116–20. 10.1111/j.1365-2133.2011.10776.x 22182184

[243] Malik M Gürcan HM Ahmed AR. Coexistence of mucous membrane pemphigoid and connective-tissue disease. Clin Exp Dermatol (2010) 35(2):156–9. 10.1111/j.1365-2230.2009.03222.x 19438545

[244] Zakka LR Reche PA Ahmed AR The molecular basis for the presence of two autoimmune diseases occurring simultaneously – preliminary observations based on computer analysis. Autoimmunity (2012) 45(3):253–63. 10.3109/08916934.2011.632454 22053914

[245] Shipman AR Reddy H Wojnarowska F. Association between the subepidermal autoimmune blistering diseases linear IgA disease and the pemphigoid group and inflammatory bowel disease: two case reports and literature review. Clin Exp Dermatol (2012) 37(5):461–8. 10.1111/j.1365-2230.2012.04383.x 22712854

[246] Takegami Y Makino T Matsui K Ueda C Fukuda S Hashimoto T Coexistence of antilaminin-332-type mucous membrane pemphigoid, lamina lucida-type linear IgA bullous dermatosis and Sjögren syndrome. Clin Exp Dermatol (2013) 38(2):194–6. 10.1111/ced.12030 23397948

[247] Monshi B Groth S Richter L Schmidt E Zillikens D Rappersberger K. A long-term study of a patient with anti-p200 pemphigoid: correlation of autoantibody levels with disease activity and an example of epitope spreading. Br J Dermatol (2012) 167(5):1179–83. 10.1111/j.1365-2133.2012.11076.x 22639938

[248] Yamada H Nobeyama Y Matsuo K Ishiji T Takeuchi T Fukuda S A case of paraneoplastic pemphigus associated with triple malignancies in combination with antilaminin-332 mucous membrane pemphigoid. Br J Dermatol (2012) 166(1):230–1. 10.1111/j.1365-2133.2011.10520.x 21777224

[249] Ohata C Higashi Y Yamagami J Koga H Ishii N Kanekura T Coexistence of pemphigus herpetiformis with IgG antibodies to desmocollin 1 and pemphigoid with IgG antibodies to BP180 C-terminal domain and laminin γ2. JAMA Dermatol (2013) 149(4):502–4. 10.1001/jamadermatol.2013.1916 23715433

[250] Li X Qian H Ishii N Yamaya M Fukuda H Mukai H A case of concurrent antilaminin γ1 pemphigoid and antilaminin-332-type mucous membrane pemphigoid. Br J Dermatol (2014) 171(5):1257–9. 10.1111/bjd.13107 25262782

[251] Kaune KM Kasperkiewicz M Tams D Bergmann M Zutt M Anti-p200/anti-laminin γ1 pemphigoid and BP180 NC16A/4575-positive mucous membrane pemphigoid: late diagnosis in a patient with disease-related loss of vision and multiple previous surgical interventions. Hautarzt (2015) 66(1):60–4. 10.1007/s00105-014-3529-1 25339385

[252] Swanton J Isenberg D. Mixed connective tissue disease: still crazy after all these years. Rheum Dis Clin North Am (2005) 31:421–36. 10.1016/j.rdc.2005.04.009 16084316

[253] Cowan CG Lamey PJ Walsh M Irwin ST Allen G McKenna KE. Linear IgA disease (LAD): immunoglobulin deposition in oral and colonic lesions. J Oral Pathol Med (1995) 24(8):374–8. 10.1111/j.1600-0714.1995.tb01202.x 7500294

[254] Chan LS Soong HK Foster CS Hammerberg C Cooper KD. Ocular cicatricial pemphigoid occurring as a sequela of Stevens-Johnson syndrome. JAMA (1991) 266(11):1543–6. 10.1001/jama.1991.03470110089038 1880886

[255] De Rojas MV Dart JK Saw VP. The natural history of Stevens Johnson syndrome: patterns of chronic ocular disease and the role of systemic immunosuppressive therapy. Br J Ophthalmol (2007) 91(8):1048–53. 10.1136/bjo.2006.109124 17314145 PMC1954786

[256] Mignogna MD Fortuna G Leuci S Stasio L Mezza E Ruoppo E. Lichen planus pemphigoides, a possible example of epitope spreading. Oral Surg Oral Med Oral Pathol Oral Radiol Endod (2010) 109(6):837–43. 10.1016/j.tripleo.2009.12.044 20382044

[257] Fania L Giannico MI Fasciani R Zampetti A Ambrogio S Balestrazzi E Ocular mucous membrane pemphigoid after Lyell syndrome: occasional finding or predisposing event? Ophthalmology (2012) 119(4):688–93. 10.1016/j.ophtha.2011.09.038 22197436

[258] Sarret Y Reano A Nicolas JF Su H Thivolet J. Bullous pemphigoid and cicatricial pemphigoid: immunoblotting detection of involved autoantigens. Autoimmunity (1989) 2(2):145–53. 10.3109/08916938909019951 2491598

[259] Shannon JF Mackenzie-Wood A Wood G Goldstein D Cicatricial pemphigoid in non-Hodgkin’s lymphoma. Intern Med J (2003) 33(8):396–7. 10.1046/j.1445-5994.2003.t01-1-00430.x 12895176

[260] Aisa Y Mori T Nakazato T Yamazaki R Yamagami J Amagai M Cicatricial pemphigoid of the oropharynx after allogeneic stem cell transplantation for relapsed follicular lymphoma. Int J Hematol (2005) 82(3):266–9. 10.1532/IJH97.05061 16207603

[261] Takahara M Tsuji G Ishii N Dainichi T Hashimoto T Kohno K Mucous membrane pemphigoid with antibodies to the beta (3) subunit of Laminin 332 in a patient with acute myeloblastic leukemia and graft-versus-host disease. Dermatology (2009) 219(4):361–4. 10.1159/000243807 19797892

[262] Mahmood S Lim ZY Benton E du Vivier A Bhogal B Mufti GJ Mucous membrane pemphigoid following reduced intensity conditioning allogeneic haematopoietic SCT for biphenotypic leukaemia. Bone Marrow Transplant (2010) 45(1):195–6. 10.1038/bmt.2009.98 19430504

[263] Masunaga K Toyoda M Kokuba H Takahara M Ohyama B Hashimoto T Mucous membrane pemphigoid with antibodies to the β3 subunit of laminin 332. J Dermatol (2011) 38(11):1082–4. 10.1111/j.1346-8138.2010.01185.x 21453313

[264] Hirakawa Y Oiso N Ishii N Koga H Tatebayashi M Uchida S Mucous membrane pemphigoid with IgG autoantibodies to the 120-kDa ectodomain of type XVII collagen (BP180/linear IgA dermatosis antigen) in a patient with idiopathic thrombocytopenic purpura. Acta Derm Venereol (2015) 95(4):493–4. 10.2340/00015555-1964 25178495

[265] Endo Y Tsuji M Shirase T Fukuda S Hashimoto T Miyachi Y Angioimmunoblastic T-cell lymphoma presenting with both IgA-related leukocytoclastic vasculitis and mucous membrane pemphigoid. Eur J Dermatol (2011) 21(2):274–6. 10.1684/ejd.2010.1227 21489903

[266] Okada R Yamaguchi Y Sawaki H Hashimoto T Aihara M Development of mucous membrane pemphigoid with antibodies to the β3 subunit of laminin 332 and bronchiolitis obliterans in a patient with chronic graft-versus-host disease. Eur J Dermatol (2015) 25(5):505–6. 10.1684/ejd.2015.2622 26243509

[267] Young AL Bailey EE Colaço SM Engler DE Grossman ME. Anti-laminin-332 mucous membrane pemphigoid associated with recurrent metastatic prostate carcinoma: hypothesis for a paraneoplastic phenomenon. Eur J Dermatol (2011) 21(3):401–4. 10.1684/ejd.2011.1360 21527374

[268] Zillikens D Caux F Mascaro JM Wesselmann U Schmidt E Prost C Autoantibodies in lichen planus pemphigoides react with a novel epitope within the C-terminal NC16A domain of BP180. J Invest Dermatol (1999) 113(1):117–21. 10.1046/j.1523-1747.1999.00618.x 10417629

[269] Sekiya A Kodera M Yamaoka T Iwata Y Usuda T Ohzono A A case of lichen planus pemphigoides with autoantibodies to the NC16a and C-terminal domains of BP180 and to desmoglein-1. Br J Dermatol (2014) 171(5):1230–5. 10.1111/bjd.13097 24813536

[270] Joly P Maho-Vaillant M Prost-Squarcioni C Hebert V Houivet E Calbo S First-line rituximab combined with short-term prednisone versus prednisone alone for the treatment of pemphigus (Ritux 3): a prospective, multicentre, parallel-group, open-label randomised trial. Lancet (2017) 389(10083):2031–40. 10.1016/S0140-6736(17)30070-3 28342637

[271] Ellebrecht CT Bhoj VG Nace A Choi EJ Mao X Cho MJ Reengineering chimeric antigen receptor T cells for targeted therapy of autoimmune disease. Science (2016) 353(6295):179–84. 10.1126/science.aaf6756 27365313 PMC5343513

[272] Serr I Fürst RW Achenbach P Scherm MG Gökmen F Haupt F Type 1 diabetes vaccine candidates promote human Foxp3(+) Treg induction in humanized mice. Nat Commun (2016) 7:10991. 10.1038/ncomms10991 26975663 PMC4796321

[273] Mikecz K Glant TT Markovics A Rosenthal KS Kurko J Carambula RE An epitope-specific DerG-PG70 LEAPS vaccine modulates T cell responses and suppresses arthritis progression in two related murine models of rheumatoid arthritis. Vaccine (2017) 35(32):4048–56. 10.1016/j.vaccine.2017.05.009 28583308 PMC5568759

[274] Lutterotti A Yousef S Sputtek A Stürner KH Stellmann JP Breiden P Antigen-specific tolerance by autologous myelin peptide-coupled cells: a phase 1 trial in multiple sclerosis. Sci Transl Med (2013) 5(188):188ra75. 10.1126/scitranslmed.3006168 23740901 PMC3973034

[275] Hammers CM Chen J Lin C Kacir S Siegel DL Payne AS Persistence of anti-desmoglein 3 IgG (+) B-cell clones in pemphigus patients over years. J Invest Dermatol (2015) 135(3):742–9. 10.1038/jid.2014.291 25142730 PMC4294994

[276] Di Zenzo G Zambruno G. Clonal analysis of B-cell response in pemphigus course: toward more effective therapies. J Invest Dermatol (2015) 135:651–4. 10.1038/jid.2014.499 25666671

[277] Ellebrecht CT Payne AS. Setting the target for pemphigus vulgaris therapy. JCI Insight (2017) 2(5):e92021. 10.1172/jci.insight.92021 28289723 PMC5333961

[278] Chen J Zheng Q Hammers CM Ellebrecht CT Mukherjee EM Tang HY Proteomic analysis of pemphigus autoantibodies indicates a larger, more diverse, and more dynamic repertoire than determined by B cell genetics. Cell Rep (2017) 18(1):237–47. 10.1016/j.celrep.2016.12.013 28052253 PMC5221611

[279] Acha-Orbea H Mitchell DJ Timmerman L Wraith DC Waldor MK Tausch GS Limited heterogeneity of T cell receptors from lymphocytes mediating autoimmune encephalomyelitis allows specific immune intervention. Cell (1988) 54:263–73. 10.1016/0092-8674(88)90558-2 2455603

[280] Brocke S Gijbels K Allegretta M Ferber I Piercy C Blankenstein T Treatment of experimental encephalomyelitis with a peptide analogue of myelin basic protein. Nature (1996) 379:343–5. 10.1038/379343a0 8552189

[281] Amagai M Tsunoda K Suzuki H Nishifuji K Koyasu S Nishikawa T. Use of autoantigen-knockout mice in developing an active autoimmune disease model for pemphigus. J Clin Invest (2000) 105(5):625–31. 10.1172/JCI8748 10712434 PMC292455

[282] Sitaru C Chiriac MT Mihai S Büning J Gebert A Ishiko A Induction of complement-fixing autoantibodies against type VII collagen results in subepidermal blistering in mice. J Immunol (2006) 177(5):3461–8. 10.4049/jimmunol.177.5.3461 16920988

[283] Takahashi H Kouno M Nagao K Wada N Hata T Nishimoto S Desmoglein 3-specific CD4+ T cells induce pemphigus vulgaris and interface dermatitis in mice. J Clin Invest (2011) 121(9):3677–88. 10.1172/JCI57379 21821914 PMC3163963

[284] Hirose M Recke A Beckmann T Shimizu A Ishiko A Bieber K Repetitive immunization breaks tolerance to type XVII collagen and leads to bullous pemphigoid in mice. J Immunol (2011) 187(3):1176–83. 10.4049/jimmunol.1100596 21705619

[285] Hurskainen T Kokkonen N Sormunen R Jackow J Löffek S Soininen R Deletion of the major bullous pemphigoid epitope region of collagen XVII induces blistering, autoimmunization, and itching in mice. J Invest Dermatol (2015) 135(5):1303–10. 10.1038/jid.2014.443 25310407

